# Van Allen Probes Observations of Symmetric Stormtime Compressional ULF Waves

**DOI:** 10.1029/2021JA030115

**Published:** 2022-02-12

**Authors:** Kazue Takahashi, Chris Crabtree, A. Y. Ukhorskiy, Alexander Boyd, Richard E. Denton, Drew Turner, Matina Gkioulidou, Massimo Vellante, Harlan E. Spence

**Affiliations:** ^1^ The Johns Hopkins University Applied Physics Laboratory Laurel MD USA; ^2^ Naval Research Laboratory Washington DC USA; ^3^ Department of Space Science Aerospace Corporation Chantilly VA USA; ^4^ Department of Physics and Astronomy Dartmouth College Hanover NH USA; ^5^ Department of Physical and Chemical Sciences University of L’Aquila L’Aquila Italy; ^6^ Consorzio Area di Ricerca in Astrogeofisica L’Aquila Italy; ^7^ Institute for the Study of Earth, Oceans, and Space, University of New Hampshire Durham NH USA

**Keywords:** compressional ULF waves, inner magnetosphere, geomagnetic storms, symmetric mode structure, Van Allen Probes, gradient driven instability

## Abstract

Previous spacecraft studies showed that stormtime poloidal ultralow‐frequency (ULF) waves in the ring current region have an antisymmetric (second harmonic) mode structure about the magnetic equator. This paper reports Van Allen Probes observations of symmetric ULF waves in the postnoon sector during a moderate geomagnetic storm. The mode structure is determined from the presence of purely compressional magnetic field oscillations at the equator accompanied by strong transverse electric field perturbations. Antisymmetric waves were also detected but only very late in the recovery phase. The symmetric waves were detected outside the plasmasphere at *L* = 3.0–5.5 and had peak power at 4–10 mHz, lower than the frequency of the local fundamental toroidal standing Alfvén wave. During the wave events, the flux of protons was enhanced at energies below ∼5 keV, which appears to be a prerequisite for the waves. The protons may provide free energies to waves through drift resonance instability or drift compressional instability, which occur in the presence of radial gradients of plasma parameters.

## Key Points

- Symmetric compressional ULF (4–10 mHz) waves were excited at *L* = 3.0–5.5 during a geomagnetic storm
- The waves were associated with enhancement of the flux of protons at energy <5 keV
- Drift compressional instability is a possible wave generation mechanism

## 1 Introduction

Ultralow‐frequency (ULF) waves in the Pc4–5 band (period = 45–600 s) with strong radial and compressional magnetic field components (compressional poloidal waves) are observed at *L* > 5 within the ring current region (*L* = 3–8) during geomagnetic storms (Anderson et al., 1990; Barfield & McPherron, 1972; Lanzerotti et al., 1969; Le et al., 2017; Sonnerup et al., 1969; Takahashi et al., 1985). Similar waves are observed at large distances (*L* > 8) even when the geomagnetic activity is low (Constantinescu et al., 2009; Nishida et al., 1997; Takahashi et al., 1990; Vaivads et al., 2001; Zhu & Kivelson, 1991). Although these waves are believed to be excited by ion‐driven instabilities (e.g., Chen & Hasegawa, 1991; Southwood, 1976), they can also interact with electrons. For example, the waves may heat electrons (Lanzerotti et al., 1969), transport them radially (Ukhorskiy et al., 2009), or drive them into the atmospheric loss cone (Rae et al., 2018).

**Figure 1:**
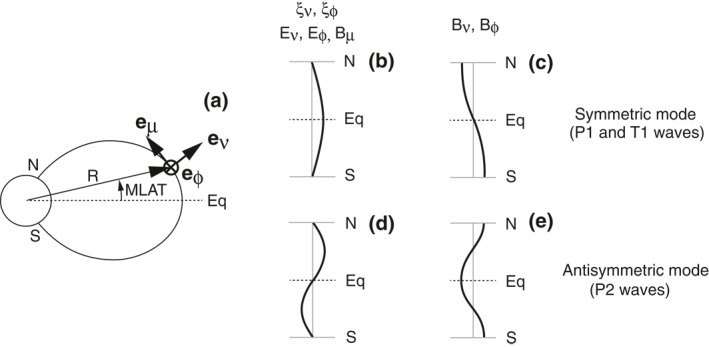
Illustration of standing waves on the background magnetic field. (a) Coordinate system. (b) Structure of the *ξ*_*ν*_, *ξ*_*ϕ*_, *E*_*ν*_, *E*_*ϕ*_, and *B*_*μ*_ components of symmetric waves. The fundamental mode is considered. (c) Structure of the *B*_*ν*_ and *B*_*ϕ*_ components of symmetric waves. Fundamental toroidal (T1) and poloidal (P1) waves have this structure. (d, e) same as (b, c) but for antisymmetric waves. The second harmonic wave is considered. Second harmonic poloidal (P2) waves have this structure.

An important factor in studying the excitation of compressional Pc4–5 waves and the interaction of the waves with charged particles is the mode structure of the waves along the background magnetic field (e.g., Southwood, 1976). ULF waves are subjected to the ionospheric boundary condition. As a consequence, the waves commonly establish a standing wave structure along the background magnetic field. The structure dictates what type of resonance is possible between the waves and particles that are executing drift and bounce motion. Figure 1 illustrates the mode structure of standing waves, using a locally defined orthogonal magnetic field aligned (MFA) coordinate system for the wave‐induced perturbations. The three axes in the MFA system are labeled *ν* (outward), *ϕ* (eastward), and *μ* (field aligned). We use the symbols *ξ*, **E**, and **B** for the field line displacement, electric field, and magnetic field, respectively.

Assuming that the background **B**‐field and plasma parameters are symmetric about the magnetic equator, we can classify the standing waves into symmetric (Figures 1b and 1c) and antisymmetric (Figures 1d and 1e) modes in terms of the pattern of *ξ* about the magnetic equator. The observable field components, *E*_*ν*_, *E*_*ϕ*_, and *B*_*μ*_ have the same symmetry as *ξ*, whereas *B*_*ν*_ and *B*_*ϕ*_ have the opposite symmetry. In general, waves with lower mode numbers carry higher energies, so the highest wave power is usually found in the fundamental (symmetric) or second (antisymmetric) harmonic mode. The fundamental and second harmonic poloidal waves, denoted P1 and P2 waves, are examples of the symmetric and antisymmetric modes observed in the magnetosphere. The illustration indicates that it is straightforward to determine the symmetry of a standing wave with a spacecraft located at the equator. If the wave has a symmetric structure, the spacecraft will detect perturbations in *E*_*ν*_, *E*_*ϕ*_, and *B*_*μ*_ (antinode) but not in *B*_*ν*_ or *B*_*ϕ*_ (node). If the wave has an antisymmetric structure, the detectable and undetectable field components are switched.

Theory indicates that the transverse and compressional components of ULF waves are generally coupled. In magnetohydrodynamic (MHD) wave theory, the inhomogeneity of the magnetosphere couples the transverse (Alfvén) waves and the compressional (fast mode) waves except at the limit of *m* = 0 (Radoski & Carovillano, 1966) or ∣*m*∣ = *∞* (Radoski, 1967), where *m* is the azimuthal wave number defined positive (negative) for eastward (westward) propagation. In a kinetic approach, Crabtree and Chen (2004) showed that the compressional component is coupled to the shear Alfvén component, as found in our case, except when the frequency is well below the shear Alfvén frequency.

Previous studies have shown that instabilities in the ring current preferentially excite antisymmetric waves. Studies of stormtime Pc5 waves observed by geostationary satellites indicated that the waves had a second harmonic (antisymmetric) structure (Le et al., 2017; Takahashi et al., 1987). Antisymmetric waves are excited during quiet times as well (W. Liu et al., 2013). These observations have been explained by mechanisms such as the drift bounce resonance instability (Southwood, 1976) and drift Alfvén ballooning instability (Chen & Hasegawa, 1991).

Symmetric poloidal standing waves do occur in the ring current region, but observations of these waves are mostly limited to quiet times. The most extensively studied symmetric waves are P1 waves (Dai et al., 2015; Motoba et al., 2015; Takahashi et al., 2011; Takahashi, Claudepierre, et al., 2018; Yamamoto et al., 2018). P1 waves exhibit sinusoidal waveforms and are considered to be excited through drift resonance of energetic (∼100 keV) ions (Thompson & Kivelson, 2001) in the presence of an inward gradient of the phase space density of the resonant ions (Dai et al., 2015; Takahashi, Claudepierre, et al., 2018; Yamamoto et al., 2018). Similar symmetric poloidal waves were detected by the Cluster spacecraft, but their association with ground pulsation was not discussed, and no conclusion was drawn on how the waves were excited (Eriksson et al., 2005, 2006). P1 waves are the source of giant pulsations (Pgs) observed on the ground (Takahashi et al., 2011; Takahashi, Claudepierre, et al., 2018; Yamamoto et al., 2018). Pgs are observed at *L* ∼6 over a wide range of magnetic local time (MLT; 01–18 hr) at times of low geomagnetic activity (Brekke et al., 1987; Motoba et al., 2015).

This study reports observation of symmetric stormtime compressional Pc4–5 waves by Van Allen Probes (Radiation Belt Storm Probes, RBSP) in the dayside magnetosphere. These waves have peak power at *L* < 5 and exhibit more irregular waveforms than quiet‐time P1 waves. Being excited in a region of strong magnetic field, the ion pressure is not high enough to drive the waves through the drift mirror instability (DMI). We suggest that the waves are instead driven by a gradient‐driven instability such as drift resonance instability (DRI) or drift compressional instability (DCI).

The remainder of this study is as follows. Section 2 describes experiments and data. Section 3 presents an overview of wave observations. Section 4 describes the properties of the waves. Section 5 presents a brief review of possible wave excitation mechanisms. Section 6 presents the instability analysis. Section 7 examines the electron response to the waves. Section 8 presents the discussion, and Section 9 presents the conclusions.

## 2 Experiments and Data

Data used in this study mainly come from the RBSP A and B spacecraft (Mauk et al., 2012). The data include **E**‐field (Wygant et al., 2013); **B**‐field (Kletzing et al., 2013); electron density (*n*_e_) determined from plasma wave spectra (Kurth et al., 2015); ion differential energy flux measured at 0.985 eV to 51.8 keV by the Helium, Oxygen, Proton, and Electron (HOPE) mass spectrometer (Funsten et al., 2013) and at 44.7–598 keV by the Radiation Belt Storm Probes Ion Composition Experiment (RBSPICE; Mitchell et al., 2013), and electron differential energy flux measured at 33 keV to 4.1 MeV by the Magnetic Electron Ion Spectrometer (MagEIS; Blake et al., 2013). We exclude RBSPICE data at the lowest energy 44.7 keV because of a high noise level. We use **E**‐field data that are obtained by first applying the spinfit method to the two components measured in the spacecraft spin plane and then by using the **E**⋅**B** = 0 assumption to derive the spin axis component (Wygant et al., 2013). The data have a time resolution of the spacecraft spin period (∼11 s). As for the magnetic field, we use 1‐s samples or its smoothed version (11‐s running averages) sampled at the time stamps of the **E**‐field data.

We rotate the **E** and **B** vectors into MFA coordinates (Figure 1a). For the **E**‐field, the *μ* axis is the direction of the measured magnetic field averaged over the spin period. For the **B**‐field, we use two versions of the MFA system. One uses the T89c magnetic field model (Tsyganenko, 1989) to define the *μ* axis. This model is more realistic than the dipole field but does not incorporate the ring current effect in any specific way. We intentionally use the model to see the effects of the ring current on the background magnetic field, as we discuss in Section 4.1. The other, used for spectral analysis, defines the *μ* axis for a short (e.g., 15 min) data segment by the trend magnetic field **B**_trend_ that is obtained by fitting a polynomial to the three components of the observed magnetic field (Takahashi & Denton, 2021; Zhu & Kivelson, 1994), with the degree of the polynomial set to four. In this system, the *B*_*ν*_ and *B*_*ϕ*_ components are transverse perturbations about **B**_trend_, and the compressional perturbation is given by *B*_*μ*_ − ∣**B**_trend_∣. For wave analysis, the **E**‐field data are also detrended by subtraction of a polynomial trend function from each component.

The spacecraft data are supplemented by data from the European Quasi‐Meridional Magnetometer Array (EMMA; Lichtenberger et al., 2013). During the time interval studied, 24 EMMA magnetometers were in operation spanning *L* = 1.57–6.49. We use the standard *H* (horizontal northward) and *D* (horizontal eastward) coordinate system for the ground magnetic field data. We also use solar wind OMNI data and geomagnetic activity indices AU, AL, and Dst.

## 3 Overview of Wave Observation

**Figure 2:**
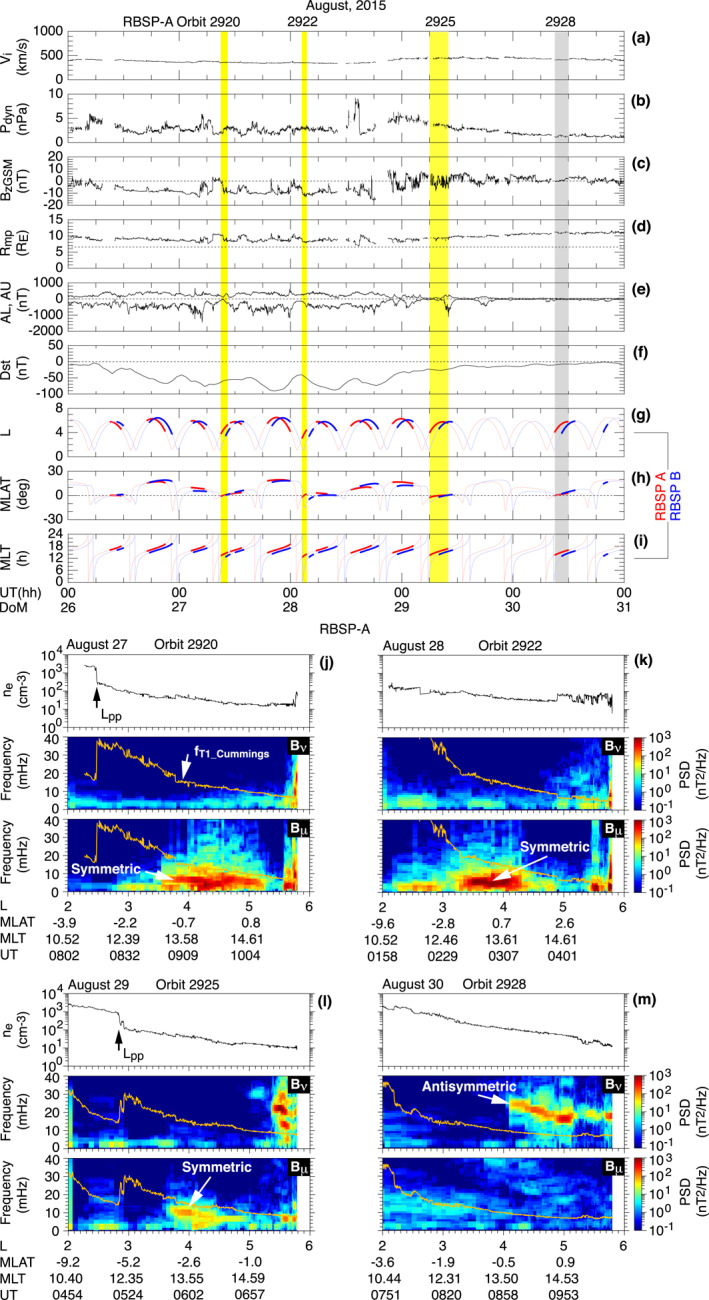
Overview of observation of ULF waves by RBSP A and B on August 26–30, 2015. (a–c) Solar wind parameters selected from the OMNI data. (d) Magnetopause standoff distance according to Shue et al. (1998). The horizontal dashed line indicates the maximum *L* (6.5) reached by the RBSP spacecraft. (e, f) Geomagnetic activity indices. (g–i) Dipole coordinates of RBSP A (red) and B (blue). The colored thick portion of the coordinate plots indicates detection of poloidal Pc4–5 waves. The yellow or gray shading highlights observations by RBSP A near the magnetic equator on its outbound orbit legs. (j–m) Electron number density and the power spectra of the *B*_*ν*_ and *B*_*μ*_ components on the four highlighted orbit legs of RBSP A, plotted as a function of *L*. The power spectra are computed using a 15‐min data window, which is moved forward in 2.5 min steps. The orange traces in the bottom two panels labeled *f*_T1_Cummings_ indicate the model fundamental toroidal wave frequency described in Section 4.2.

Figure 2 presents an overview of the space environment and magnetospheric ULF waves on August 26–30, 2015. The top three panels show solar wind parameters taken from the OMNI data provided in 1 min resolution: the ion bulk velocity *V*_i_, the dynamic pressure *P*_dyn_, and the *z* component of the magnetic field in geocentric solar magnetospheric coordinates *B*_*z*GSM_. The velocity is moderate at 400–500 km/s and shows smooth and slow variations. In contrast, *P*_dyn_ varies significantly between 0.9 and 9.2 nPa. Most importantly, *B*_*z*GSM_ varies between −16 and 10 nT with negative values dominating from midday on August 26 until the end of August 28. This *B*_*z*GSM_ behavior explains the ∼2 days interval spanning August 26–29 of repeated enhancements of the auroral electrojet according to the AL and AU indices (Figure 2d) and a moderately developed ring current according to the Dst values between −100 and −40 nT (Figure 2f). The Dst index does not follow a simple pattern of a storm: a single main phase (Dst decrease) and a single recovery phase (Dst increase). Instead, we see quasiperiodic variations with minima occurring ∼12 hr apart.

Figures 2g–2i show the locations of RBSP A (red) and RBSP B (blue) in dipole coordinates: geocentric equatorial distance of the field line that passes the spacecraft (*L*), magnetic latitude (MLAT, in degrees), and MLT (in hr). The thick line segments in these figures indicate the time intervals of poloidal ULF waves in the Pc4–5 band (1.7–22 mHz) that are identified by visual inspection of time series plots and dynamic spectra of the **E** and **B** fields measured by the spacecraft. By “poloidal,” we mean substantial perturbations in at least two of the *E*_*ϕ*_, *B*_*ν*_, and *B*_*μ*_ components. On some orbits, the wave detection occurred very close to the magnetic equator (∣MALT∣ < 3°), which enables us to distinguish between symmetric and antisymmetric waves from their node and antinode signatures at the equator (Figure 1). The shading in Figures 2a–2i highlights four such equatorial observations with RBSP A, which occurred on the outbound leg of orbits 2920, 2922, 2925, and 2928. On the first three of these orbits, symmetric waves (shaded yellow) were detected. On the last orbit, antisymmetric waves (shaded gray) were detected.

We determined the symmetry of the waves using the magnetic field power spectral density (PSD) shown in Figures 2j–2m, plotted as a function of *L*. We include *n*_e_ at the top and indicate the location of the outer edge of the electron plasmapause (*L*_PP_) by an arrow directed upward. On the first orbit (2920), plasmapause crossing occurred at *L* = 2.5. On the second orbit (2922), the plasmapause was apparently located at *L* < 2.1. On the third orbit (2925), the crossing occurred at *L* = 2.9. By the time of the fourth orbit (2928), the plasmapause either had been smoothed out or had moved beyond spacecraft apogee (*L* = 5.8).

Included in each spectrogram is the model fundamental toroidal (T1) wave frequency labeled *f*_T1_Cummings_. We obtained *f*_T1_Cummings_ by solving the toroidal wave equation of Cummings et al. (1969) for a dipole magnetic field assuming that the mass density (*ρ*) varies along the magnetic field line as 1/*r*, where *r* is geocentric distance. Use of the dipole field is justified because we are interested in the region inward of *L* = 5, where the dipole field is strong, and the geomagnetic storm was not very strong according to the Dst values shown in Figure 2f. Following previous magnetoseismic studies (e.g., Takahashi et al., 2006), we introduce the average ion mass *M*_i_ (≡*ρ*/*n*_e_) and express the mass density as *ρ* = *n*_e_ *M*_i_. We set *M*_i_ at 3 amu following the statistical result obtained using data from the Combined Release and Radiation Effects Satellite (CRRES; Takahashi et al., 2006). We adopted the same *M*_i_ value for all orbits shown in Figure 2.

The *B*_*ν*_ and *B*_*μ*_ PSD shown in the second and third panels of Figures 2j–2m demonstrates changes in wave properties through the course of the storm. The spectrograms for the first three orbits show strong *B*_*μ*_ power peaked at 4–10 mHz within *L* = 3–5.5. This frequency is well below *f*_T1_Cummings_. There is no corresponding power enhancement in the *B*_*ν*_ component. We take the absence of *B*_*ν*_ power as evidence for an equatorial node of that component because the spacecraft was virtually on the equator during these wave events. We conclude that symmetric waves were detected on the first three orbits because symmetric waves have an equatorial node of *B*_*ν*_ and an equatorial antinode of *B*_*μ*_.

We see different waves on the fourth orbit (Figure 2m). The waves appear at *L* = 4.1–5.8 only in the *B*_*ν*_ spectrogram, and their frequency is ∼2*f*_T1_, falling steadily as *L* increases. The strong *B*_*ν*_ component and the absence of *B*_*μ*_ perturbation indicate that the waves had an antisymmetric structure. Both the frequency and the polarization indicate that the waves are P2 waves, which are commonly excited in the ring current region (Cummings et al., 1969). Recent RBSP studies (Min et al., 2017; Oimatsu et al., 2018; Takahashi, Oimatsu, et al., 2018; Yamamoto et al., 2019) presented detailed analysis of the physical properties and the generation mechanism of P2 waves. Z. Y. Liu et al. (2020) studied the ion response to the P2 waves reported by Yamamoto et al. (2019).

## 4 Wave Properties

**Figure 3:**
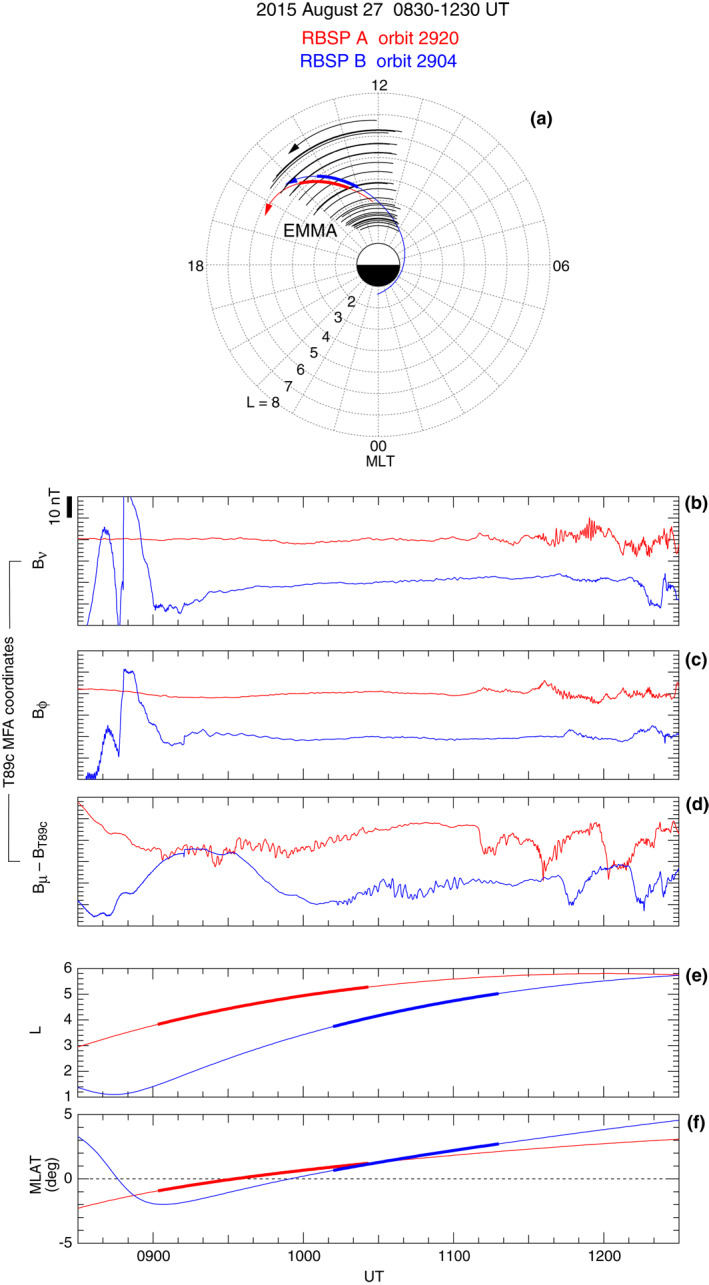
Magnetic field observation by RBSP A (red traces) and B (blue traces) at 0830–1230 UT on August 27, 2015. (a) Observatory positions in *L*‐MLT coordinates. The thick portions of the RBSP orbits indicate detection of compressional Pc4–5 oscillations. The black arcs indicate the 24 EMMA stations. (b)–(d) Magnetic field components in the local MFA coordinate system based on the T89c model field (Tsyganenko, 1989). Vertical offsets are added to separate data from the two spacecraft. (e, f) *L* and MLAT of the spacecraft. The heavy portion of the line plots indicates the time interval of the compressional Pc4–5 oscillations discussed in the text.

We examine the symmetric compressional waves in detail, focusing on observations made on August 27, 2015 by RBSP A (orbit 2920), RBSP B (orbit 2904), and EMMA. Figure 3a shows the locations of the spacecraft and the EMMA magnetometers at 0830–1230 UT in *L*‐MLT polar coordinates. The two spacecraft had nearly identical orbits, with RBSP B trailing RBSP A by ∼70 min. The EMMA stations covered the *L* and MLT positions of the spacecraft very well. The heavy portions of the spacecraft orbit traces indicate where the symmetric waves were detected.

### 4.1 Localization in *L*

The waves are localized in *L*. Figures 3b–3d compare the magnetic field data from RBSP A and RBSP B. The MFA coordinate system for this figure is based on the T89c model (Tsyganenko, 1989), with Figure 3d showing the difference between the *B*_*μ*_ component and the magnitude of the T89c field. The median values of the field components are removed, and ±10 nT offsets are added to separate data from the two spacecraft. The spacecraft positions in *L* and MLT are also plotted (Figures 3e and 3f). The magnetic field data indicate that the waves of interest to us produce only compressional (*B*_*μ*_) perturbations.

Figure 3d indicates a time delay of the compressional wave event between the two spacecraft. RBSP A detected the waves at 0902–1026 UT, which corresponds to the *L* range 3.8–5.3 (Figure 3e, heavy line). RBSP B detected the event ∼1 hr later at 1012–1128 UT, but in a very similar *L* range, 3.7–5.0. During the time interval of overlap (1012–1026 UT), when the spacecraft were separated ∼1.3 in *L* and ∼0.2 hr in MLT, the waveform differs between the two spacecraft. These features imply that the compressional waves are localized in *L* and had a coherence length shorter than 1.3 *R*_E_.

The compressional oscillations are detected in a region where the observed *B*_*μ*_ is depressed relative to the T89c model. The depression, which has a magnitude of ∼20 nT, appears because the model does not represent the effects of stormtime ring current. The collocated wave activity and magnetic field depression imply that the waves were excited by an instability associated with elevated plasma pressure.

### 4.2 Frequency and Polarization

**Figure 4:**
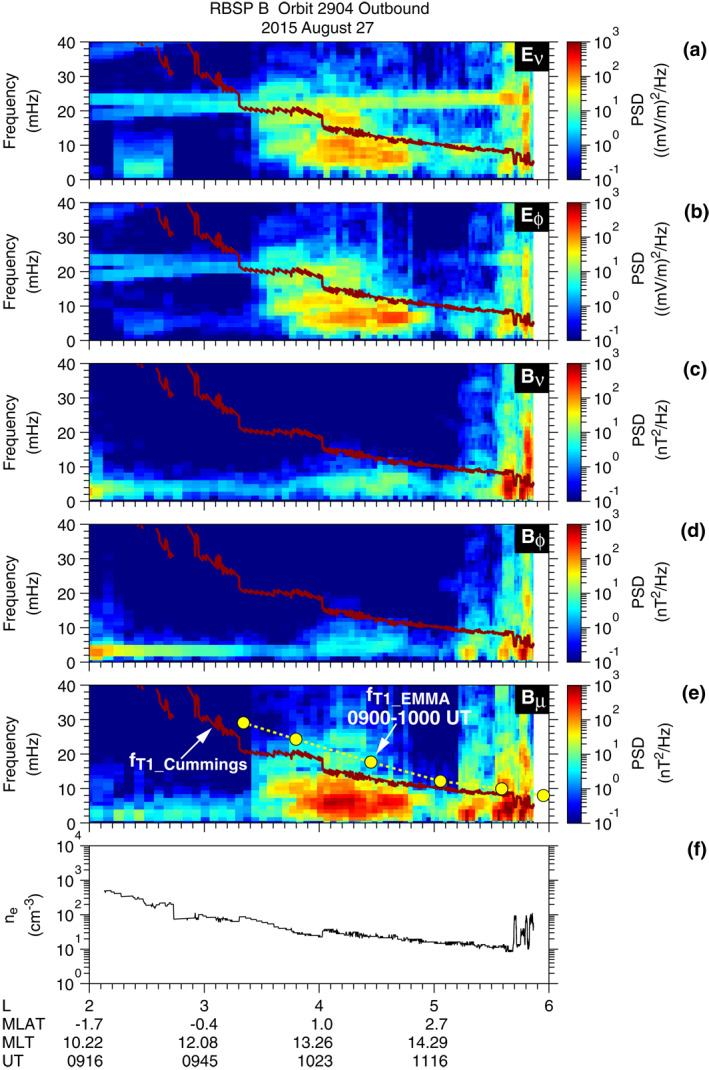
(a)–(e) *L* versus frequency plots of the power spectral density of the full **E**‐field and **B**‐ field components at RBSP B on the outbound leg of orbit 2904. *f*_T1_Cummings_ is plotted in brown in each panel. The yellow filled circles in panel (e) indicate the fundamental toroidal wave frequency obtained using the cross‐phase analysis of EMMA data. They are plotted as a function of the *L* value of the EMMA stations defined using the T02 model. (f) Electron density.

Figure 4 shows frequency versus *L* spectrograms for all field components at RBSP B on the outbound leg of orbit 2904. The *B*_*ν*_ and *B*_*μ*_ spectrograms are very similar to those at RBSP A (Figure 2j) regarding both frequency (∼7 mHz) and localization in *L* (3.5–5.0). Both *E*_*ν*_ and *E*_*ϕ*_ exhibit power spectra very similar to those of *B*_*μ*_. The strong **E**‐field power is what we expect for symmetric waves. There is only weak power in the *B*_*ϕ*_ component consistent with symmetric waves observed near the magnetic equator (∣MLAT∣ < 3°).

To address the question of whether the compressional waves are strongly coupled to standing Alfvén waves (e.g., Chen & Hasegawa, 1991), we estimated the frequency of fundamental toroidal waves (denoted *f*_T1_) by applying the cross‐phase analysis technique (Waters et al., 1991) to EMMA data for 0900–1000 UT. We chose this time interval because the EMMA stations were close to 12 hr MLT and the cross‐phase signatures are clear at many stations. The yellow filled circles labeled “*f*_T1_EMMA_” in Figure 4e indicate the estimated frequency. The *L* values of the EMMA stations are defined using the T02 model (Tsyganenko, 2002). These *L* values are larger than those calculated using the International Geomagnetic Reference Field (IGRF) model (by ∼0.1 at *L* ≃ 3 and by ∼0.7 at *L* ≃ 6) because of the ring current effect (Berube et al., 2006). We find that *f*_T1_EMMA_ decreases monotonically from 24 mHz at *L* = 3.6–11 mHz at *L* = 5.1 and is well above the center frequency ∼7 mHz of the compressional waves. We also examined EMMA data for 1010–1110 UT, which corresponds to the time interval of the symmetric waves at RBSP B (Figure 3d), and found *f*_T1_EMMA_ at *L* > 4 was very close to that for 0900–1000 UT. Because EMMA did not fully cover the magnetic field footprint of the spacecraft, we also show *f*_T1_Cummings_, which is obtained using the *n*_e_ data (Figure 4f) as described in Section 3. It is obvious that the compressional waves are not coupled to T1 waves on the local field line. However, we cannot exclude the possibility of coupling of the symmetric waves to P1 waves. The P1 frequency (*f*_P1_) is lower than *f*_T1_ according to cold plasma MHD theory (Cummings et al., 1969), and *f*_P1_ can be even lower if there is a radial gradient of the ring current thermal pressure (R. E. Denton et al., 2003).

**Figure 5:**
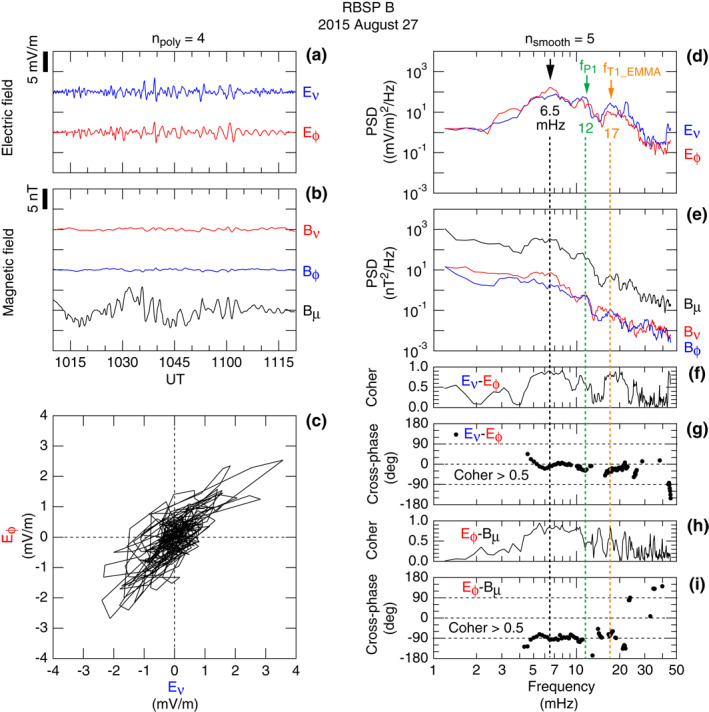
Time and frequency domain properties of compressional waves detected at RBSP B at 1010–1220 UT on August 27, 2015. The time series is detrended by subtraction of a polynomial as described in the text. The spectra are smoothed by 5‐point averaging. (a) Detrended electric field. (b) Detrended magnetic field. (c) Electric field hodogram. (d) Power spectra of the electric field components. The highest peak occurs at 6.5 mHz and is marked by a black dashed line. The orange dashed line marks the frequency (17 mHz) of another spectral peak found in multiple field components, which coincides with *f*_T1_EMMA_ shown in Figure 4e. The fundamental poloidal wave frequency *f*_P1_ (= 12 mHz) is estimated by multiplying *f*_T1_EMMA_ by the theoretical ratio *f*_P1_/*f*_T1_ ∼0.7 (Cummings et al., 1969). (e) Power spectra of the magnetic field components. (f, g) Coherence and cross‐phase spectra computed from the *E*_*ν*_ and *E*_*ϕ*_ time series. (h, i) Coherence and cross‐phase spectra computed from the *E*_*ϕ*_ and *B*_*μ*_ time series.

Figure 5 shows the temporal and spectral properties of the compressional waves observed by RBSP B on orbit 2904. The spacecraft was located at *L* = 4.5 at the center of the time interval shown. The **E**‐field time series indicates that the waves had comparable toroidal (*E*_*ν*_) and poloidal (*E*_*ϕ*_) components with the amplitudes reaching 5 mV/m peak‐to‐peak (Figure 5a). Magnetic field oscillations appear mostly in the *B*_*μ*_ component with a maximum amplitude of 6 nT peak‐to‐peak (Figure 5b). The *E*_*ν*_‐*E*_*ϕ*_ hodogram (Figure 5c) shows highly elliptical polarization, with the major axis of polarization tilted ∼45° from the *ν*‐axis.

The symmetric waves produce a broad peak at ∼6.5 mHz in the PSD of the *E*_*ν*_, *E*_*ϕ*_, and *B*_*μ*_ components (Figures 5d and 5e). Additional spectral peaks appear at 12 and 17 mHz, the latter of which coincides with the frequency (the orange downward arrow and vertical dashed line) estimated by interpolating the *f*_T1_EMMA_ data points shown in Figure 4e to *L* = 4.5. The frequency 12 mHz at the second spectral peak matches *f*_P1_ that is obtained by multiplying *f*_T1_EMMA_ by the theoretical *f*_P1_/*f*_T1_ ratio of ∼0.7 (Cummings et al., 1969). We can conclude that the dominant symmetric waves had a frequency much lower than the frequency of the cold plasma fundamental toroidal and poloidal waves excited on the same field line. The *E*_*ν*_‐*E*_*ϕ*_ coherence is high (Figure 5f) and the *E*_*ν*_‐*E*_*ϕ*_ cross‐phase is ∼0 (Figure 5g) in the band occupied by the symmetric waves, meaning that these two components oscillate in phase. The *E*_*ϕ*_‐*B*_*μ*_ coherence is also high in the wave band (Figure 5h), and the *E*_*ϕ*_‐*B*_*μ*_ cross‐phase is ∼− 90° (Figure 5i), meaning that the time average of the radial component of the Poynting flux is zero and the wave energy does not propagate radially. This suggests that the mode is radially bound. This is also consistent with the observations that the fluctuations are localized in *L*.

### 4.3 Relation to Magnetic Pulsations on the Ground

**Figure 6:**
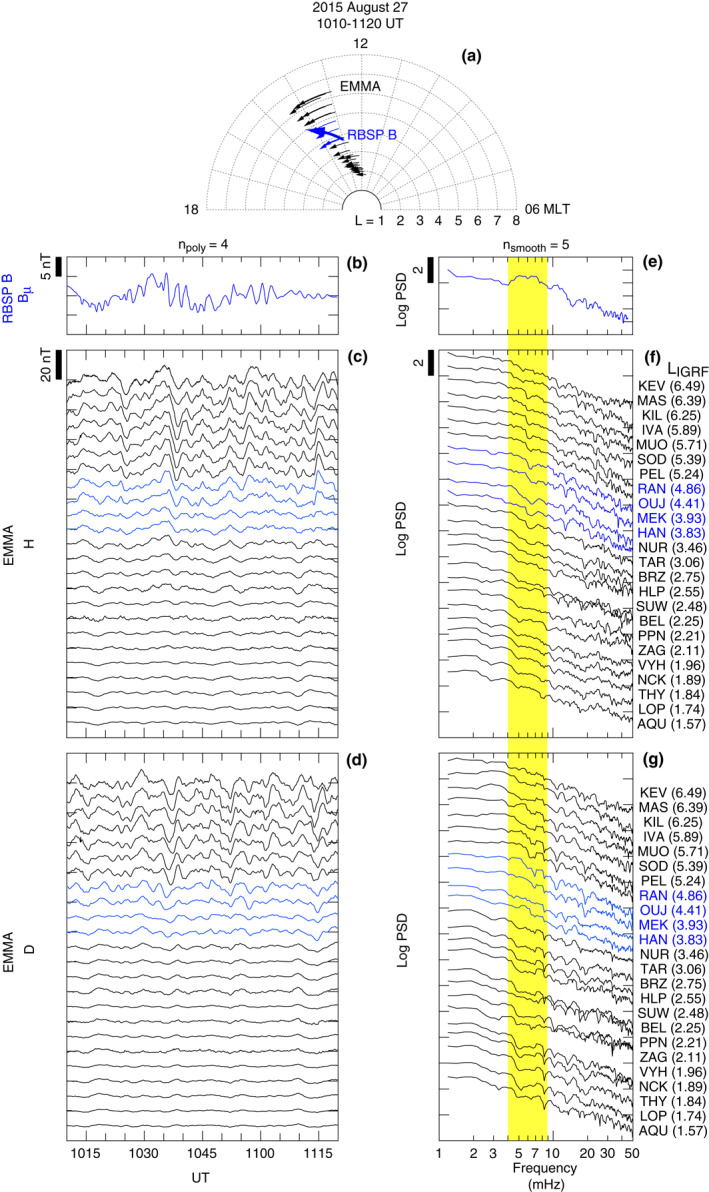
Comparison of compressional magnetic field oscillations at RBSP B and ground magnetic field oscillations at EMMA. EMMA data for the *L* range (3.7–5.0) of the waves observed by RBSP B are shown in blue. (a) Locations of the spacecraft and EMMA magnetometers for the selected time interval 1010–1120 UT on August 27, 2015. (b)–(d) Magnetic field time series for (b) RBSP B, (c) EMMA station *H* component, (d) and EMMA station *D* component. (e)–(g) Power spectra computed from the detrended time series. The station code and *L* value of the EMMA stations are shown on the right of the spectrum stack plots.

Figure 6 shows a comparison of the *B*_*μ*_ oscillations at RBSP B and magnetic field oscillations detected by EMMA at 1010–1120 UT on August 27. The spacecraft and ground magnetometers were in good conjunction in *L* and MLT during this 70 min interval, as shown in Figure 6a. We can gain information on the azimuthal scale size or the azimuthal wave number (*m*) of the symmetric waves from the space‐ground comparison. If the waves have a horizontal scale size at ionosphere height (∼100 km) that is comparable to or shorter than the height, they cannot be detected with ground magnetometers (Hughes & Southwood, 1976; Yeoman et al., 2000). This screening effect explains why magnetospheric ULF waves are detected by ground magnetometers only when ∣*m*∣ ≤ 50 (Takahashi et al., 2013; Wright & Yeoman, 1999; Yamamoto et al., 2018).

In the waveform plots (Figures 6b–6d), we find little similarity between space and ground, and in the spectrum plots (Figures 6e–6g), we do not see any outstanding peaks on the ground in the band occupied by the compressional waves in space (4–9 mHz, shaded yellow). The implication is that the symmetric waves had large (>50) ∣*m*∣ values corresponding to azimuthal wavelength of <4,000 km at the magnetic equator when mapped along the dipole field lines at *L* = 4.5. However, it is also possible that the waves were highly localized to the equatorial region in the magnetosphere with negligible coupling to the Alfvén mode, in which case there will be no detectable wave fields either at the ionosphere or on the ground regardless of the magnitude of *m*.

The oscillations on the ground are similar at different locations, so large‐scale (∣*m*∣ < 10) *P*_dyn_ variations in the solar wind are the most likely driver of the oscillations. At RBSP B, the *P*_dyn_‐driven oscillations were probably masked by the internally excited symmetric waves.

## 5 Possible Wave Excitation Mechanisms

In this section, we examine instabilities that may be relevant to the observed symmetric waves. Source mechanisms external to the magnetosphere are excluded based on the observations indicating that the waves are localized in *L* (Figure 3) and inferred to have large azimuthal wave numbers (Figure 6). The depression of the background magnetic field during the wave events (Figure 3) also points to internal instabilities associated with enhanced particle pressure.

### 5.1 Drift Resonance Instability

Symmetric poloidal standing Alfvén waves with a finite compressional component can be excited through drift resonance

(1) $$\omega -m\omega_{d}=0,$$

where *ω* is the wave frequency and *ω*_d_ is the bounce averaged magnetic field gradient and curvature drift frequency of particles. We refer to this mechanism as DRI. We consider ring current ions and westward‐propagating waves for this resonance. Theory predicts that the phase of particle flux oscillation changes by ∼180° across the resonance energy (Kivelson & Southwood, 1985) and that the resonance transfers energy from ions to poloidal waves in the presence of an inward gradient of the phase space density (F) of the ions (Chen & Hasegawa, 1991; Southwood, 1976)

(2) $${\left.\frac{\partial F\left(L,M,J\right)}{\partial L}\right|}_{M_{\text{res}},J_{\text{res}}}<0,$$

where *M* (magnetic moment) and *J* are the first and second adiabatic invariants of particle motion with *M*_res_ and *J*_res_ being the values for the particles that are in resonance with the waves.

Symmetric poloidal waves and their association with DRI have been reported. Some of the P1 wave studies mentioned in Section 1 (Dai et al., 2013; Takahashi, Claudepierre, et al., 2018; Yamamoto et al., 2018) confirmed the phase shift of ion flux oscillations across the resonance energy and found an inward phase space density gradient. These P1 waves were all observed at geomagnetically quiet times. However, we cannot exclude the possibility that DRI excites symmetric poloidal waves during geomagnetic storms.

We note that particle resonance condition with symmetric waves is generally expressed as

(3) $$\omega -m\omega_{d}=N\omega_{b},$$

where *N* is an even integer and *ω*_b_ is the particle bounce frequency (Southwood & Kivelson, 1982). The resonance given by Equation 1 is a special case (*N* = 0) of this condition. We consider the *N* = 0 resonance here with the assumption that the lowest‐order resonance contributes most efficiently to the energy exchange between waves and particles. Although the *N* = 0 resonance have been reported for P1 waves as stated above, we are aware that energy exchange can occur through *N* ≠ 0 resonances. For a complete evaluation of wave growth, resonance terms with all *N* values need to be considered. Also, even when we consider only the *N* = 0 resonance, a complete instability analysis would include evaluation of the *L* gradient of different *J*_res_ values corresponding to different equatorial pitch angles. In previous studies, only limited *J*_res_ values (e.g., *J*_res_ ≃ 0, corresponding to ∼90° equatorial pitch angle) have been considered.

### 5.2 Drift Mirror Instability

The DMI was proposed as a mechanism to generate stormtime compressional ULF waves (Lanzerotti et al., 1969). The condition for the instability is

(4) $$\tau \equiv 1+\sum_{j}\beta_{⊥j}\left(1-\frac{P_{⊥j}}{P_{∥j}}\right)<0,$$

where *j* indicates ion species, *β* is the ratio of the thermal to magnetic pressures, *P* is the particle pressure, and the symbols ⊥ and ∥ indicate directions perpendicular and parallel to the background magnetic field, respectively. The initial DMI theory (Hasegawa, 1969) did not take into account the dipole magnetic field geometry or the effect of wave reflection at the ionosphere. Chen and Hasegawa (1991) updated the theory, including the dipole geomagnetic field, coupling of DMI to field line eigenmodes (standing Alfvén waves), and ion bounce motion, and suggested that the coupled drift Alfvén ballooning mirror (DABM) instability excites antisymmetric waves.

In a recent study using RBSP B data, Soto‐Chavez et al. (2019) reported that DMI excited a magnetically compressional ULF wave at ∼7 mHz. The wave was observed at *L* > 5 near the magnetic equator (MLAT ≃ − 0.7°), in the evening sector, and during the main phase of a moderate geomagnetic storm (Dst minimum = − 79 nT). The value of $\beta_{H^{+}}$ obtained using RBSPICE data was high (>1). No analysis was presented on the mode structure (symmetric or antisymmetric) along the background magnetic field.

It is possible that the wave reported by Soto‐Chavez et al. (2019) had an antisymmetric structure. The wave had a considerable *δB*_*ν*_ component unlike our symmetric waves but similar to the antisymmetric stormtime Pc5 waves reported by Takahashi et al. (1987), where the symbol *δ* indicates a perturbation. But the equatorial node of antisymmetric waves can be shifted away from the dipole equator when the dipole tilt angle is large, especially on the nightside. The tilt angle during the Soto‐Chavez et al. (2019) event was large (∼30°), and we suspect that the equatorial node of the wave was not located exactly at the dipole equator (MLAT = 0). In fact, the TS05 magnetic field model (Tsyganenko & Sitnov, 2005) places both the minimum magnetic field magnitude and the field line maximum distance at MLAT = − 2.2° for the field line that passes the spacecraft at the epoch of the maximum wave activity (1405 UT on July 6, 2014). If the wave symmetry can be defined with respect to this modified equator, the spacecraft was located 1.5° north of the node. In this case, simultaneous detection of *δB*_*μ*_ and *δB*_*ν*_ of an antisymmetric wave is possible according to Figures 3 and 5 of Takahashi et al. (1987), and the DABM instability is a viable excitation mechanism.

### 5.3 Drift Compressional Instability

Compressional magnetospheric waves are excited by an instability when the radial gradients of plasma bulk parameters satisfy certain conditions. We refer to this instability as the DCI. DCI theory was initially developed using a slab geometry for the plasma and magnetic field (Hasegawa, 1971a, 1971b). Rosenbluth (1981) discussed the instability mechanism incorporating the effects of trapped particles. Ng et al. (1984) considered trapped particles and bounce averaging in a dipole magnetic field and used a symmetric trial function to study the instability. Cheng and Lin (1987) studied both DMI and DCI without setting the field line mode structure a priori and showed that the former has an antisymmetric structure whereas the latter has a symmetric structure. Crabtree et al. (2003) studied DCI in realistic magnetic fields in the drift‐kinetic approximation and showed that there are two types of instabilities, which are described below. Crabtree and Chen (2004) added finite Larmor radius effects and performed a rigorous analysis of how the compressional modes decoupled from the electrostatic and shear Alfvén modes. P. N. Mager et al. (2013) studied the effect of a bump‐on‐tail ion energy distribution function on DCI.

Some observational studies considered DCI as a possible source mechanism for ULF waves. C. A. Green (1985) suggested DCI for plasmaspheric giant pulsations observed on the ground. More recently, the instability was considered for symmetric poloidal waves observed by the Cluster spacecraft in the dayside magnetosphere at *L* = 4–6 during a geomagnetically quiet period (Eriksson et al., 2005), compressional Pc5 waves observed by the five Time History of Events and Macroscale Interactions during Substorms Mission spacecraft at *L* ≃ 10 in the dusk sector (Rubtsov et al., 2018), and nighttime Pc5 waves detected by radar (Chelpanov et al., 2016; Chelpanov et al., 2018; O. V. Mager et al., 2019). One of the radar events, which was detected at *L* = 4.6–7.8 in the postmidnight sector at ∼1.8 mHz and exhibited *m* ≃ − 10, was also detected by RBSP (O. V. Mager et al., 2019). It is questionable that this radar event was of the same origin as our symmetric waves because our waves had higher frequencies and likely much larger ∣*m*∣.

We provide a brief review of key DCI properties obtained by Crabtree et al. (2003) and Crabtree and Chen (2004) that are relevant to our spacecraft observations. Although their studies were motivated by Pi2 waves excited on the nightside, the results can be applied to dayside ULF waves as well. The theory assumed hot ions (∼10 keV protons) to be the energy source.

DCI occurs in two situations. The first is when the pressure gradients become sufficiently steep to reverse the magnetic‐guiding center drift (referred to as DCI condition 1). In the dayside inner magnetosphere where the magnetic field is dominated by the dipole component, the ion magnetic field gradient and curvature drift are westward. Therefore, for ions to satisfy DCI condition 1 (westward diamagnetic drift), the gradient of the ion pressure (*P*) needs to be outward,

(5) ∂P/∂L>0.

The second situation is when the ion temperature (*T*) gradient is in the opposite direction to the ion density (*n*) gradient (referred to as DCI condition 2). This condition can be expressed as

(6) $$\eta \equiv L_{n}/L_{T}<0,$$

where $L_{n}$ (≡ (*∂* log *n*/*∂L*)^−1^) and $L_{T}$ (≡ (*∂* log *T*/*∂L*)^−1^) are the density and temperature gradient scale lengths with the sign included. For fixed *η*, there is a range of *β*_low_ < *β* < *β*_high_ that is unstable, and for a fixed *β*, there is a range of *η*_1_ < *η* < *η*_2_ < 0 that is unstable. For *η*_*i*_ < ∼0.5, both *β*_low_ and *β*_high_ become smaller for more negative *η*_*i*_ (see Figure 1 of Crabtree and Chen (2004) for the region in parameter space for instability).

The basic understanding of the physical mechanism of instability for the DCI was first given by Rosenbluth (1981) and is similar to the physics that drives the mirror modes (Southwood & Kivelson, 1993). Here, we give a short analogy of DCI to DMI. At low frequencies, the perturbed particle pressure *δP*_⊥_ is a function of the magnitude of the magnetic field perturbation *δB*_*μ*_. In a homogeneous anisotropic plasma, relevant to DMI, the perturbed pressure for the bulk of the particles is given as

(7) $$\delta P_{⊥bulk}≃2P_{⊥}\left(1-T_{⊥}/T_{\|}\right)\delta B_{\mu }/B,$$

where *B* is the magnitude of the magnetic field (Southwood & Kivelson, 1993).

However, resonant particles, with zero parallel velocity for purely growing modes, are different, and they respond in such a way that the perturbed pressure is in phase with the magnetic perturbation for growing waves. In fact, it is possible to express the resonant particle contribution to the perturbed pressure as

(8) $$\delta P_{⊥res}≃2\frac{\gamma }{k_{\|}}n_{\text{res}}\frac{T_{⊥}^{2}}{T_{\|}}\frac{\delta B_{\mu }}{B},$$

where *γ* is the growth rate, *k*_‖_ is the wavenumber parallel to the magnetic field, and *n*_res_ is the density of resonant particles. Then the change in energy due to a perturbation near marginal stability is

(9) $$\delta W_{\text{DMI}}≃\left\{\frac{B^{2}}{\mu_{0}}+2P_{⊥}\left(1-\frac{T_{⊥}}{T_{\|}}\right)+2\frac{\gamma }{k_{\|}}n_{\text{res}}\frac{T_{⊥}^{2}}{T_{\|}}\right\}\frac{\delta B_{\mu }}{B}≃0.$$

To maintain pressure balance when *T*_⊥_/*T*_‖_ > 1, where the bulk of the particles are responding out of phase with the magnetic field, the perturbed magnetic field must be growing (*γ* > 0).

DCI is similar except that the perturbed pressure of the nonresonant particles is now given as

(10) $$\delta P_{⊥bulk}≃\left(\partial P/\partial L\right){\left(\partial B/\partial L\right)}^{-1}\delta B_{\mu }.$$

Then if the product of the two *L* derivatives on the right‐hand side of Equation 10 is negative, which means that the magnetic and pressure perturbations are out of phase, we see that the pressure gradients and magnetic gradients in physical space play the same role as the temperature anisotropy in mirror modes. The resonant particles in the DCI are resonant with the bounce‐averaged magnetic guiding center drift of the ions (rather than the parallel motion), and there is a real frequency component to the DCI. Thus, we find an equation similar to the drift mirror modes,

(11) $$\delta W_{\text{DCI}}≃\left\{\frac{B^{2}}{\mu_{0}}+\left(\partial P/\partial L\right){\left(\partial \log\;B/\partial L\right)}^{-1}+P_{⊥res}\right\}\frac{\delta B_{\mu }}{B}≃0.$$

In Crabtree and Chen (2004), the resonant particle contribution is found by solving the gyrokinetic equation, but physically the role of the resonant particles is the same. They respond in phase to the magnetic perturbation only in growing waves, and the balancing of the total pressure leads to the instability.

Because the frequencies are low, one must consider the bounce‐averaged motion along the field lines, as was done in Crabtree and Chen (2004) and Cheng and Lin (1987). For *δB*_*μ*_ eigenmode structures that are antisymmetric around the magnetic field, the resonant particle contribution to the perturbed pressure vanishes by bounce averaging. Because DCI is a resonant particle instability, as explained above, DCI should have a symmetric eigenmode structure.

## 6 Examination of Instability Conditions

We examine whether DRI, DMI, or DCI is relevant to the symmetric waves using proton measurements made at RBSP A on orbit 2920 with the HOPE and RBSPICE instruments. These instruments detected He^+^ and O^+^ also, but we do not include these ions in our analysis because their contribution to the plasma pressure was much smaller than H^+^. To indicate that we are using only H^+^ data, we add the subscript “H^+^” to ion plasma parameters.

### 6.1 Proton Data Overview

**Figure 7:**
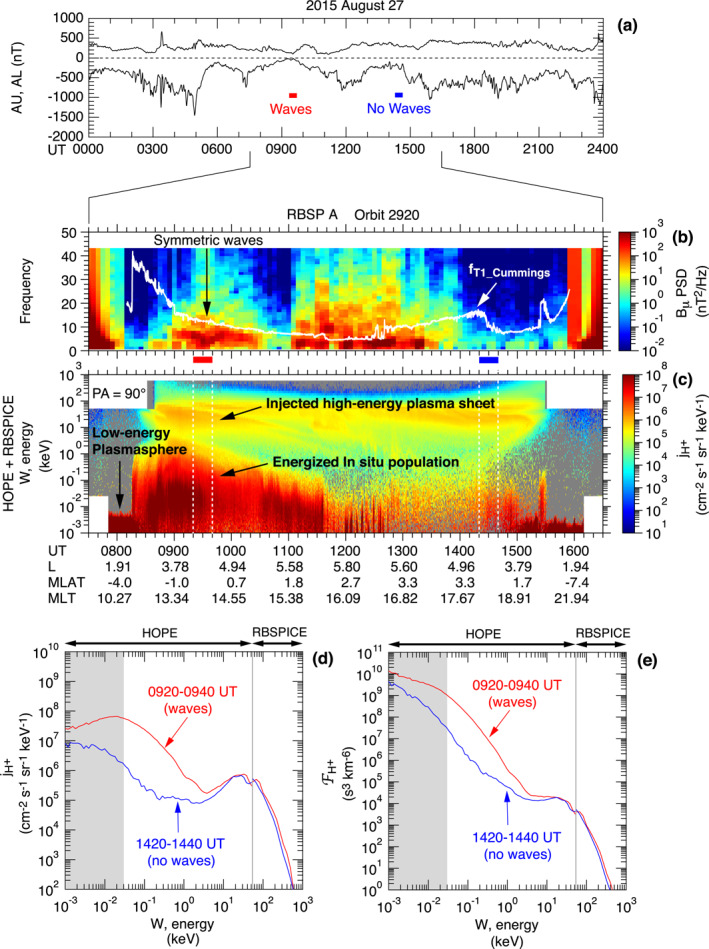
(a) Auroral electroject indices for August 27, 2015. (b) Dynamic spectra of the *B*_*μ*_ component. The red horizontal bar at the bottom indicates the 0920–0940 UT interval (*L* = 4.2–4.6) on the outbound leg when the intensity of symmetric waves was high. The blue horizontal bar indicates the time interval 1420–1440 UT on the inbound leg when the spacecraft moved from *L* = 4.6 to *L* = 4.2 but did not detect symmetric waves. These time markers are also shown in panel (a). (c) Energy‐time display of proton differential energy flux at 90° pitch angle measured by HOPE and RBSPICE. (d) Proton differential energy flux averaged over the time intervals shown by the red and blue time markers The vertical line indicates the energy boundary (∼53 keV) between HOPE and RBSPICE. The shading indicates energies (<30 eV) that are not included in calculating the proton bulk parameters shown in Figure 9. (e) Same as (d) but for the phase space density.

Figure 7 provides an overview of the proton data. The orbit occurred during a period of moderate auroral electrojet activity (Figure 7a), so injection of particles in the midnight sector is expected. The frequency‐time spectrogram of the *B*_*μ*_ component (Figure 7b) indicates symmetric waves on the outbound leg at 0900–1030 UT (*L* = 3.8–5.3). Compressional waves are detected also at large distances, from 1110 UT (*L* = 5.6) to 1320 UT (*L* = 5.4) encompassing the apogee (1200 UT, *L* = 5.8), with broader spectral bandwidths than the symmetric waves. We do not discuss the mode structure or the generation mechanism of these large‐*L* waves. No notable waves are seen at *L* < 5.3 on the inbound leg. The difference between the two orbital legs can be either temporal or spatial. Because the spacecraft crossed *L* = 4.5 at 14.1 hr MLT on the outbound leg (waves detected) and at 18.2 hr MLT on the inbound leg (no waves detected), it is possible that the waves were limited to MLTs earlier than 18 hr.

Figure 7c is an energy (*W*)‐time spectrogram of the proton differential energy flux $\left(j_{H^{+}}\right)$ at 90° pitch angle, generated by combining data included in the HOPE and RBSPICE level‐3 data products (ect‐hope‐PA‐L3 and rbspice_lev‐3‐pap_tofxeh). The protons appearing in this figure form three populations, referred to as “low‐energy plasmasphere,” “energized in situ population,” and “injected high‐energy plasma sheet,” following M. H. Denton et al. (2016). The injected population is explained by drift of plasmasheet ions that are injected from the nightside into drift orbits passing the *L* < 6 region, but the in situ population cannot be explained by this mechanism. Possible mechanisms to produce the energized in situ population include wave‐particle interaction involving ion‐acoustic waves, Alfvén waves, or ion cyclotron waves (M. H. Denton et al., 2016). The symmetric waves were detected outside the plasmasphere, so the latter two populations are of interest to us. The two populations are separated by a demarcation energy of ∼5 keV. The energized in situ population starts from the lower energy limit of the instrument (∼0.001 keV) and extends to a cutoff, which changes from ∼3 keV at 0900 UT to <0.1 keV after 1100 UT. This population exhibits high flux values and high cutoff energies when the symmetric waves are present, but it is much weaker on the outbound leg, which may explain the absence of symmetric waves. The high‐energy plasma sheet population forms narrow horizontal structures above ∼10 keV. Within this population, there is a time delay in the appearance of lower‐energy protons (energy dispersion), which is attributed to the energy dependence of the magnetic gradient and curvature drift speed of particles injected on the nightside. The high‐energy plasma sheet population is present on both the outbound and inbound legs with similar energies and intensities.

Figure 7d compares $j_{H^{+}}$ versus energy plots for the two 20 min intervals marked in Figure 7b. At *W* < ∼5 keV, $j_{H^{+}}$ is significantly higher in the first interval (0920–0940 UT, red curve) than in the second interval (1420–1440 UT, blue curve). At *W* > ∼5 keV, the difference is small. This suggests that protons at *W* < ∼5 keV play an important role in exciting the symmetric waves.

Figure 7e shows phase space density plots for the same time intervals. An important feature to note is the slope $\frac{\partial F_{H^{+}}}{\partial W}$ at *W* > 1 keV. The high‐energy population that forms a peak in the $j_{H^{+}}$ plots produces a plateau (outbound leg) or a mild positive slope (inbound leg) for $F_{H^{+}}$ (Figure 7e) in the energy range 3–20 keV. A positive slope (or a bump on tail phase space density) is a possible free energy for bounce resonance excitation of P2 waves (Hughes et al., 1978). The absence of a positive energy slope on the outbound leg implies that P2 waves were not destabilized, which is consistent with the dominance of symmetric waves.

### 6.2 Drift Resonance Instability

Assuming that the symmetric waves propagate westward and can satisfy the resonance condition given by Equation 1 at some energy, we examine whether an earthward phase space density gradient (Equation 2) was present to make symmetric waves unstable.

**Figure 8:**
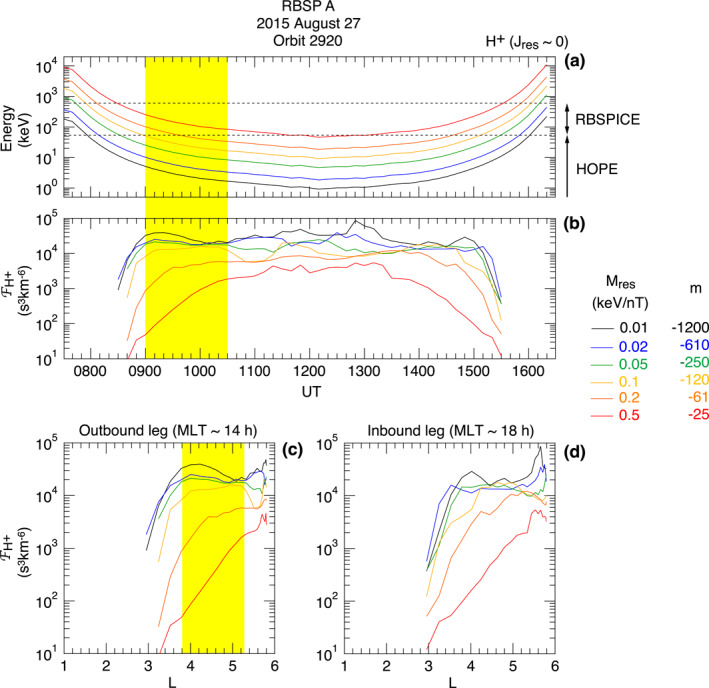
Proton parameters relevant to DRI, derived from measurements made at RBSP A on orbit 2920. (a) Proton energies corresponding to the six trial values of magnetic moment *M*_res_ defined using the magnitude of the measured magnetic field. The yellow shading indicates the time interval of symmetric waves. (b) Variation of proton phase space density $\left(F_{H^{+}}\right)$ evaluated at the selected magnetic moments. We define $F_{H^{+}}$ using 10 min averages of $j_{H^{+}}$. (c) The same $F_{H^{+}}$ values plotted as a function of dipole *L* for the outbound and inbound legs.

Figure 8 shows properties of protons that are relevant to DRI. Assuming that equatorially mirroring particles interact with the symmetric waves most effectively, we evaluate $F_{H^{+}}$ at *J*_res_ = 0. On the selected RBSP A orbit, the equatorially mirroring particles are nearly equivalent to particles having local pitch angles of ∼90° because the spacecraft was very close to the magnetic equator (∣MLAT∣ < 3.5°) at *L* > 3. Therefore, we evaluate $F_{H^{+}}$ at 90° pitch angle at the spacecraft. As for *M*_res_, we consider six trial values, 0.01, 0.02, 0.05, 0.1, 0.2, and 0.5 (keV/nT). At *L* = 4.5, where the symmetric wave was strong, the equatorial dipole field magnitude 330 nT gives the corresponding proton energies of 3.3, 6.6, 16.5, 33, 66, and 165 keV. For these energies, we use the approximate formula by Hamlin et al. (1961) to find the guiding center drift frequency $\left(\omega_{dH^{+}}\right)$ and then obtain the azimuthal wave number (*m*, negative for westward propagation) that satisfies the resonance condition (Equation 1) by setting the wave frequency at 7 mHz to represent the observation (see Figure 5). The *m* values, shown in Figure 8, vary from −1,200 for *M*_res_ = 0.01 keV/nT to −25 for *M*_res_ = 0.5 keV/nT. The ∣*m*∣ value of 1,200 is much larger than those reported for Pc5 waves (Takahashi et al., 1985; Wright & Yeoman, 1999). However, this ∣*m*∣ translates to an azimuthal wavelength of 150 km, which is still larger than the gyroradius (∼30 km) of the resonant (3.3 keV) protons.

Figure 8a shows time series of the proton energies corresponding to the trial *M*_res_ values plotted in 10 min steps. To get the energies (= *M*_res_ *B*), we used the measured *B*, which is slightly (∼10%) lower than the dipole value. The yellow shading indicates where the symmetric waves were detected. We have marked the energy limits of HOPE and RBSPICE to show that these instruments cover the six energies except near perigee. The corresponding $F_{H^{+}}$(*M*_res_, *J*_res_) time series, also given in 10 min steps, is shown in Figure 8b. There is a sharp decline in $F_{H^{+}}$ as the spacecraft approaches the perigee. Elsewhere, the behavior of $F_{H^{+}}$ depends on *M*_res_.

The same $F_{H^{+}}$ data are plotted as a function of dipole *L* for the outbound leg (Figure 8c) and the inbound leg (Figure 8d). Figure 8c indicates an inward gradient of $F_{H^{+}}$ occurring in the *L* domain of the symmetric waves for the three lowest *M*_res_ values corresponding to the resonance energy (at *L* = 4.5) of ≤16.5 keV and ∣*m*∣ ≥ 250. This result indicates that DRI is a possible mechanism for the symmetric waves. The $F_{H^{+}}$ profile for the inbound leg is not as clear, but there is an indication of an inward gradient for the three lowest *M*_res_ values. Despite the inward gradient, no symmetric waves were detected on the inbound leg. Perhaps the gradient was not steep enough or the magnitude of $F_{H^{+}}$ was not high enough to excite symmetric waves.

### 6.3 Drift Mirror Instability

**Figure 9:**
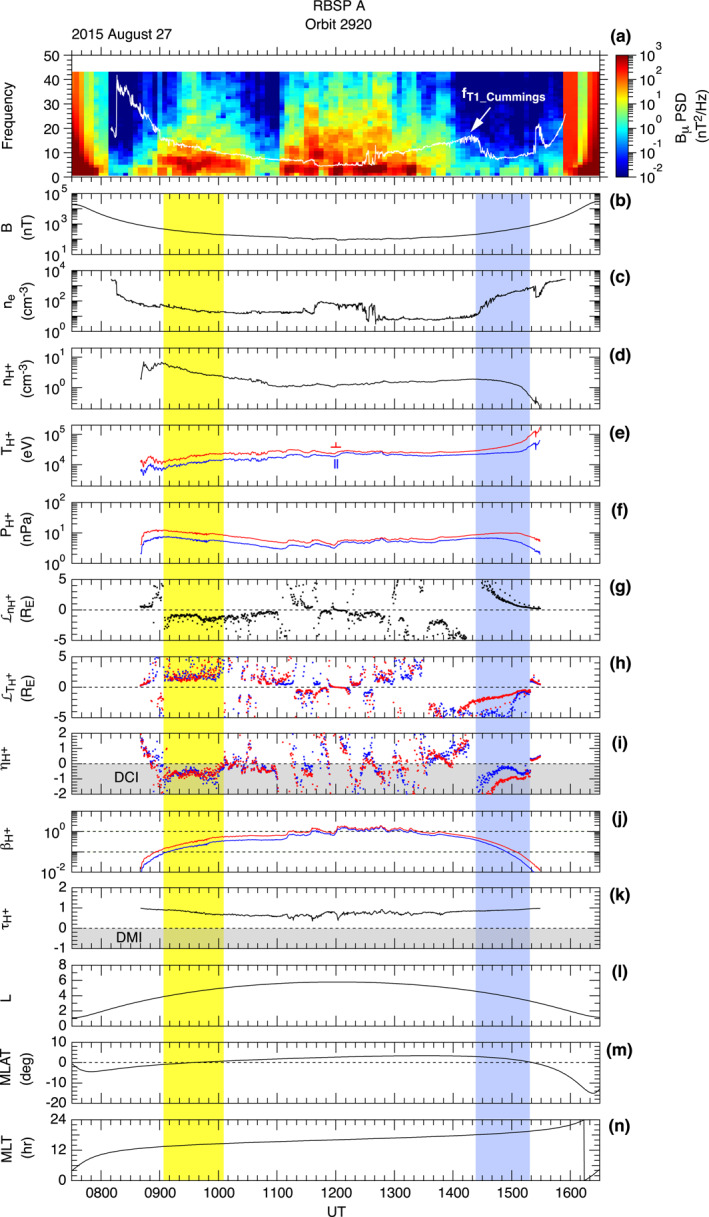
Relationship between compressional magnetic field oscillations and quantities related to DMI and DCI, shown for RBSP A orbit 2920. (a) Dynamic spectra of the *B*_*μ*_ component. (b) Magnetic field magnitude. (c) Electron number density. (d)–(k) Parameters derived from proton fluxes measured by the HOPE and RBSPICE experiments. See text for definition. Some parameters are color coded to indicate the components perpendicular (red) and parallel (blue) to the background magnetic field. The areas shaded yellow or blue indicate sustained intervals of negative $\eta_{H^{+}}$. The gray shading indicates the parameter domain satisfying the condition for DCI or DMI. (l–n) Magnetic coordinates of the spacecraft.

Figure 9 contains proton bulk parameters relevant to DMI along with the *B*_*μ*_ dynamics spectra, the magnetic field magnitude, and the electron density (top three panels) and the spacecraft position (bottom three panels). The number density ($n_{H^{+}}$, Figure 9d) and pressure ($P_{H^{+}}$, Figure 9f) are obtained from the moments of $j_{H^{+}}$ measured by HOPE and RBSPICE. The proton temperature ($T_{H^{+}}$, Figure 9e) is given by $T_{H^{+}}=P_{H^{+}}/\left(k_{B}n_{H^{+}}\right)$, where *k*_B_ is the Boltzmann constant. The moment calculation uses HOPE data in the energy range 30 eV to 51.8 keV and RBSPICE data in the energy range 54.7–598 keV. Although HOPE detects ions with energies as low as 1 eV, the moment calculation uses data for >30 eV to avoid possible errors arising from spacecraft charging.

It is clear that DMI is an unlikely mechanism for the symmetric waves. Figure 9k shows the instability parameter $\tau_{H^{+}}$ defined by Equation 4. Although the temperature anisotropy $T_{⊥H^{+}}>T_{∥H^{+}}$ (Figure 9e) is qualitatively favorable for the instability, *τ* remains >0 throughout the orbit because $\beta_{H^{+}}$ (Figure 9j) is not high enough to bring *τ* down to the instability regime (<0, shaded gray in Figure 9k).

### 6.4 Drift Compressional Instability

DCI is a more promising mechanism for the symmetric waves. Included in Figure 9 are the parameters relevant to DCI defined in Section 5.3: $L_{n_{H^{+}}}$, $L_{T_{H^{+}}}$, and $\eta_{H^{+}}$. We calculated $L_{n_{H^{+}}}$ and $L_{T_{H^{+}}}$ after smoothing the $n_{H^{+}}$ and $T_{H^{+}}$ time series by 55‐point (20 min) running average. Two time intervals of sustained negative $\eta_{H^{+}}$ are evident, and we highlight them by shading in yellow (outbound leg) and blue (inbound leg). In the time interval shaded yellow, $n_{H^{+}}$ decreased with *L* (Figure 9d), while $T_{H^{+}}$ increased (Figure 9e). The median values during the symmetric wave event are $L_{n_{H^{+}}}\;∼\;-1.3\;R_{E}$, $L_{T_{H^{+}}}\;∼$ 1.7 *R*_E_. As a result, we get $\eta_{H^{+}}\;∼\;-0.6$, which satisfies DCI condition 2 (Equation 6).

We can make a quantitative evaluation of the frequency of waves generated by DCI. First, the theory by Crabtree and Chen (2004) predicts that the wave frequency (*ω*) is on the order of the proton diamagnetic drift frequency $\left(\omega_{*H^{+}}\right)$, expressed as

(12) $$\omega_{*H^{+}}=k_{\phi }\rho_{H^{+}}v_{H^{+}}/L_{n_{H^{+}}},$$

where *k*_*ϕ*_ is the angular azimuthal wave number, $\rho_{H^{+}}$ is the proton Larmor radius, and $v_{H^{+}}$ is the proton velocity. Then including the temperature gradient into the diamagntic drift frequency and assuming the distribution is approximately Maxwellian (locally), we get

(13) $$\omega ≃\omega_{*H^{+}}\left(1+1.5\eta_{H^{+}}\right).$$

Using the $T_{⊥H^{+}}$ value of ∼15 keV (Figure 9e), we obtain $\rho_{H^{+}}$ ≃ 50 km and $v_{H^{+}}$ ≃ 1,700 km/s, and we get $|L_{n_{H^{+}}}|\;≃\;1.3\;R_{E}$ from Figure 9g. Because the remaining parameter *k*_*ϕ*_ on the right‐hand side of Equation 12 cannot be determined observationally, let us first assume ∣*m*∣ ≃ 100, which is a reasonable value according to the discussion of the ionospheric screening effect presented in Section 4.3. Then for *L* ≃ 4.5 (an approximate center location of the symmetric waves), we get *k*_*ϕ*_ ≃ 100/4.5 ≃ 22 rad $R_{E}^{-1}$. From these, we get $k_{\phi }\rho_{H^{+}}≃0.2$, a reasonable value according to Crabtree and Chen (2004), and from Equations 12 and 13, we get *ω* ≃ 0.005 rad Hz or ∼1 mHz.

The *ω* value obtained above is about an order of magnitude too small for our observations and definitely lower than the local P1 wave frequency. But if $\beta_{H^{+}}\ll \;\omega_{{\text{dH}}^{+}}/\omega_{*H^{+}}$, then the frequency is closer to the bounce averaged drift frequency of the ions. A simplified explanation for this is that if $\beta_{H^{+}}$ is small enough, then the resonant contribution has to be closer to the thermal core of the ions to get the resonant contribution in order to drive the instability. This makes the mode behave more like an “energetic particle mode” or beam‐like mode. If $\beta_{H^{+}}$ is large enough, then a small fraction of resonant particles is sufficient to drive the instability and the wave takes on the characteristic of the neutrally stable drift mode. So if $\beta_{H^{+}}≃0.5$, then $\omega_{{\text{dH}}^{+}}$ is about an order of magnitude higher to bring *ω* closer to the observed ∼7 mHz. Another way to bring theoretical *ω* closer to the observation is to assume a much larger ∣*m*∣ value, for example, ∣*m*∣ ≃ 500, in which case we have $k_{\phi }\rho_{H^{+}}≃1$. However, such a high ∣*m*∣ number has not been observed, as we pointed out in Section 6.2.

DCI condition 2 $\left(\eta_{H^{+}}<0\right)$ also occurred on the inbound leg of the same orbit at 1420–1520 UT (shaded blue), when the spacecraft was close to the magnetic equator (∣MLAT∣ < 3°, Figure 9m) and in an *L* range (2.9–4.4, Figure 9l) similar to that during the symmetric wave event on the outbound leg. However, no notable *B*_*μ*_ oscillation was detected on the inbound leg. The reason for this difference might be that the $n_{H^{+}}$ and $T_{H^{+}}$ gradients each had a sign opposite that during the first interval, the high electron density, or the low intensity of the thermal population.

In relation to the $F_{H^{+}}$ plot shown in Figure 7e, we point out that the theoretical DCI analysis (Crabtree & Chen, 2004; Crabtree et al., 2003) assumed a single Maxwellian for the ions. The shape of the observed $F_{H^{+}}$ is not a single Maxwellian, which implies that there are multiple gradient scale lengths, which would complicate stability analysis. However, we believe that the instability occurs even when more than one gradient scale exists. We argue that the theory gives a conservative estimate of the instability threshold when we use the bulk parameters derived for ions having a structured phase space density because a structured phase space would only add to the free energy available to drive the instability.

### 6.5 Caveats

Our analysis of DRI and DCI is incomplete in two aspects. First, there is ambiguity between spatial and temporal variations in the structure of the background plasma. What we defined as a radial gradient or a radial gradient scale length could have significant error due to temporal effects such as energy dispersion of ion injections or changes in solar wind dynamic pressure. For the August 27 event examined in detail, the RBSPICE data were missing from RBSP B, which makes the RBSP A and B comparison difficult.

Second, we lack some observations that are crucial to unambiguously determining the wave generation mechanism. One is the azimuthal wave number (including both the magnitude and the sign). This means that we cannot determine $k_{\phi }\rho_{H^{+}}$, an important parameter in the DCI theory. The *m* value also determines whether drift resonance (Equation 1) is possible. Another is the cross‐phase between *δB*_*μ*_ and $\delta j_{H^{+}}$, which provides a test of drift resonance. The cross‐phase will change by ∼180° across the resonance energy in association with a peak amplitude of $\delta j_{H^{+}}$ (Dai et al., 2015). Although RBSP carried instruments to detect ions at relevant energies, they were not sensitive enough to determine $\delta j_{H^{+}}$ during the selected symmetric wave events.

## 7 Electron Response

This section examines the relationship between symmetric waves and energetic electrons. ULF waves with both low‐*m* and high‐*m* numbers can interact with energetic electrons. A general theoretical description has been given by Kivelson and Southwood (1985). Mann et al. (2013) presented evidence that low‐*m* (∼2) waves can interact with energetic electrons through drift resonance. Claudepierre et al. (2013) reported electron resonance with a P1 wave having an intermediate *m* value of ∼40. Ukhorskiy et al. (2009) proposed that high‐*m* waves with a broad‐*m* spectrum can lead to substantial electron energization and radial transport. Zong et al. (2017) presented a comprehensive review on the subject.

### 7.1 Electron Flux Phase Space Density

**Figure 10:**
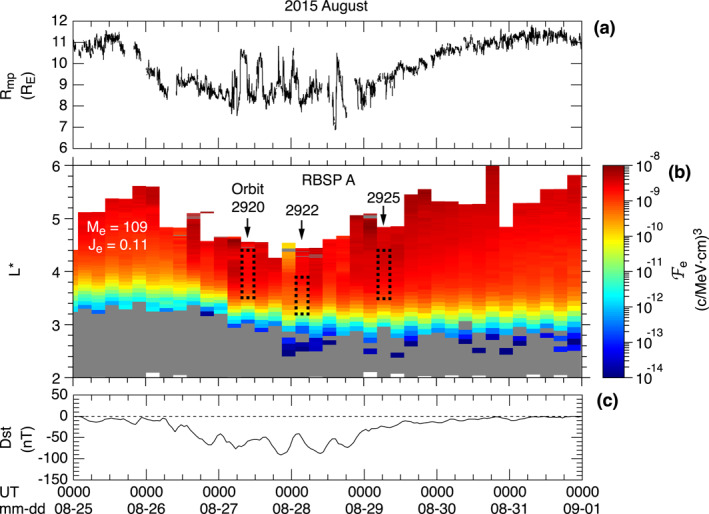
Overview of the relationship between electron phase space density and symmetric waves. (a) Magnetopause standoff distance according to Shue et al. (1998). The solar wind OMNI data are used as input. (b) Electron phase space density at RBSP A evaluated for *M*_e_ = 109 MeV/G and *J*_e_ = 0.11 *R*_E_G^1/2^ and plotted as a function of time and L*. Each pixel covers a half orbit (4.5 hr) along the time axis. The rectangles drawn with black dotted lines indicate the symmetric compressional waves identified in Figure 2. (c) Dst index.

To understand whether the symmetric waves play any role in the transport of electrons in the outer radiation belt, we first examine the spatial and temporal relationship between the waves and electrons. Figure 10 shows the evolution of the phase space density $\left(F_{e}\right)$ of energetic electrons at RBSP A (Figure 10b) along with a model magnetopause standoff distance (Figure 10a) and Dst (Figure 10c) during the selected storm interval. $F_{e}$ is obtained using the standard definition $F_{e}$ = $j_{e}/p_{e}^{2}$, where *p*_*e*_ is the electron momentum (Hilmer et al., 2000). The illustrated $F_{e}$ is evaluated at *M* = 109 MeV/G and *J* = 0.11 *R*_*E*_G^1/2^ and plotted as a function of time and L*, where L* the third adiabatic (magnetic flux) invariant.

The range of L* covered by RBSP A during the detection of symmetric waves is marked by a rectangle drawn for orbits 2920, 2922, and 2925 (see Figure 2). Here we emphasize that compressional ULF waves were detected by RBSP A on every orbit from 2917 (August 26) to 2925 (August 29), as shown in Figure 2g. It is likely that the waves had a symmetric structure on all or some of these orbits. We find that the inner L* edge of the symmetric waves is nearly colocated with the L* region where $F_{e}$ exhibits a steep outward gradient. In this region, the waves may diffuse the electron inward by violating the L* values of the electrons and move the inner edge of the radiation belt closer to Earth (Green & Kivelson, 2001).

### 7.2 Electron Flux Oscillations

Although it is beyond the scope of the present study to determine the role that the symmetric waves might play in redistributing radiation belt electrons over the entire *L* shells covered by the spacecraft, we examine the response of electrons to the waves using observation made at *L* ∼5. An important target of this analysis is drift resonance (Equation 1), which is the elementary process of energy exchange between ULF waves and electrons in the context of radiation belt dynamics (Elkington et al., 1999; Fälthammar, 1965).

Two conditions are necessary for the drift resonance to occur. First, the waves need to propagate eastward (*m* > 0), the same direction as the electron guiding center drift. Second, ∣*m*∣ needs to have an appropriate value to satisfy the resonance condition. For example, ∣*m*∣ must be in the range 4–120 for the electrons in the MagEIS energy range considered here (33 keV–1.6 MeV) to experience the resonance with a 7 mHz wave excited at *L* = 4.5, when the particle drift speeds are evaluated using the dipole magnetic field (Hamlin et al., 1961).

**Figure 11:**
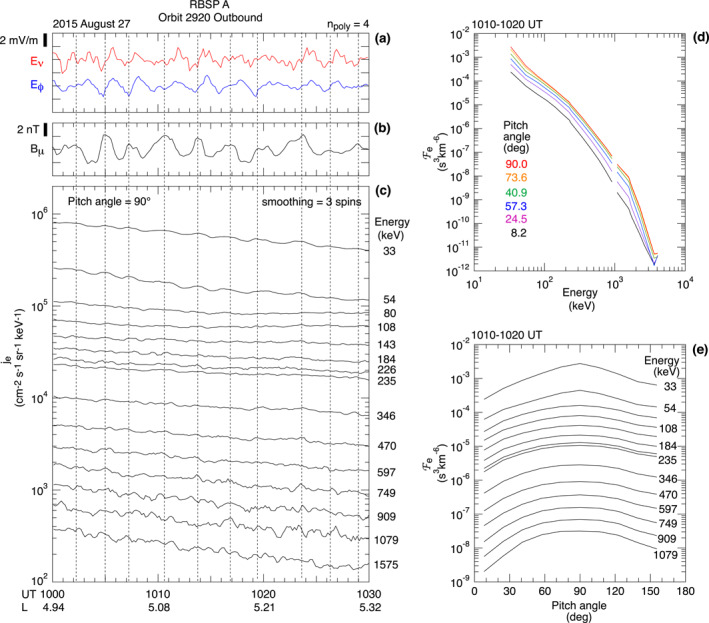
Electron flux variations during the symmetric wave event at RBSP A on orbit 2920. (a) Electric field. (b) Magnetic field compressional component. (c) Electron flux at 90° pitch angle, smoothed by averaging over three spins (33 s). Vertical dashed lines are drawn through the peaks, most clearly seen at 33 and 54 keV. (d) Energy dependence of the electron phase space density at six pitch angles averaged over 1010–1020 UT. (e) Pitch angle dependence of the electron phase space density shown at multiple energies.

We examine the temporal variation of the electron differential energy flux (denoted *j*_e_) during the wave event to gain insight into the resonance. Figure 11 shows the relationship between the symmetric waves and *j*_e_ observed at RBSP A on orbit 2920. Figures 11a and 11b show the *E*_*ν*_, *E*_*ϕ*_, and *B*_*μ*_ components of the waves, and Figures 11c shows *j*_e_ at 90° pitch angle. Oscillations are visible at all energies but most clearly at the two lowest energies (the irregular variations at high energies are attributed to low count rates). By following the vertical dashed lines drawn through the peaks of *j*_e_ at 33 keV, we find that *j*_e_ oscillates in phase with *B*_*μ*_ and ∼90° out of phase with *E*_*ν*_ and *E*_*ϕ*_.

Figure 11 includes information on the dependence of the equilibrium $F_{e}$ on *L*, pitch angle, and kinetic energy, which is necessary to determine the cause of particle flux oscillations in ULF wave fields (Kivelson & Southwood, 1985; Southwood & Kivelson, 1981). Figures 11c indicates that *j*_e_ decreased as the spacecraft moved outward. This is interpreted to be a spatial effect, that is, *j*_e_ (and $F_{e}$) had an inward gradient. Figures 11d shows that $F_{e}$ rapidly decreases as energy increases. Figures 11e shows that $F_{e}$ is peaked at 90° pitch angle.

**Figure 12:**
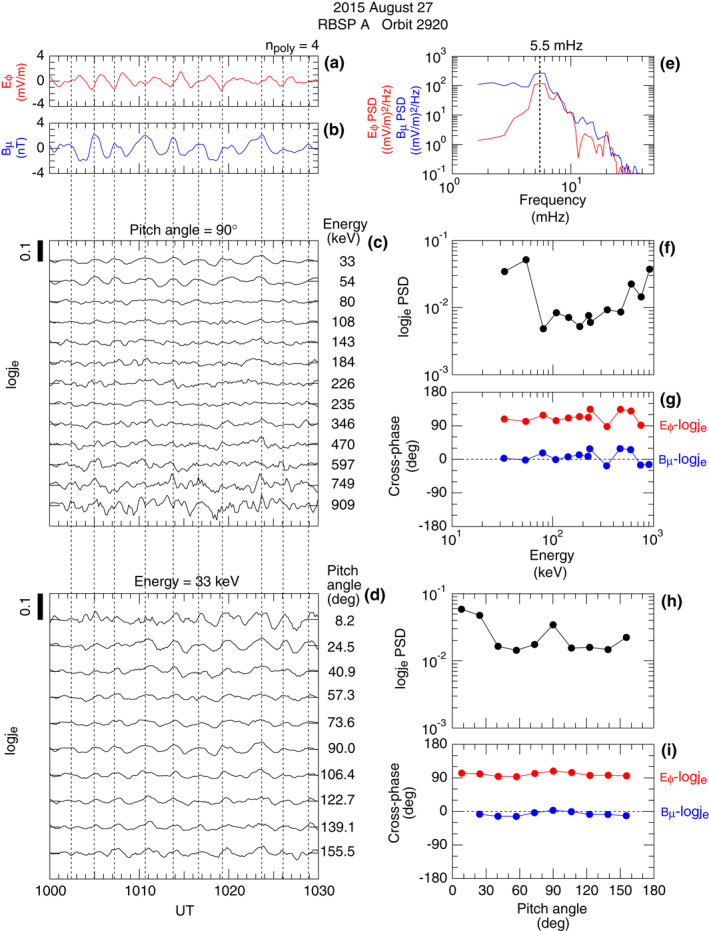
Relationship between symmetric waves and electron flux oscillations observed by RBSP A on orbit 2920. (a) Detrended *E*_*ϕ*_ time series. (b) Detrended *B*_*μ*_ time series. (c) Detrended log*j*_e_ time series at 13 energies. The pitch angle is fixed at 90°. The vertical dashed lines mark the peaks seen at 33 keV. (d) Detrended log*j*_e_ time series at 10 pitch angles. The energy is fixed at 33 keV. (e) Power spectral density (PSD) of the data shown in panels (a) and (b). The vertical dashed line marks the spectral peaks at 5.5 mHz. (f) PSD of the data shown in panel (c), evaluated at 5.5 mHz and plotted as a function of energy. (g) Cross‐phase between *E*_*ϕ*_ and log*j*_e_ (red) and between *B*_*μ*_ and log*j*_e_ (blue). (h) PSD of the data shown in panel (d), plotted as a function of pitch angle. (i) Cross‐phase between *E*_*ϕ*_ and log*j*_e_ (red) and between *B*_*μ*_ and log*j*_e_ (blue).

Figure 12 shows the detail of the *j*_e_ oscillations and their relation to the symmetric waves. We represent *j*_e_ oscillations by the detrended version of its logarithm, denoted log  *j*_e_. Figures 12a–12c indicate that log  *j*_e_ has a phase difference of ∼90° from *E*_*ϕ*_ and ∼0 from *B*_*μ*_, respectively, regardless of energy or pitch angle. This is confirmed in Figures 12g and 12i, which show the *E*_*ϕ*_ and   log  *j*_e_ spectral parameters evaluated at the wave frequency 5.5 mHz (Figure 12e). The *E*_*ϕ*_‐ log  *j*_e_ cross‐phase is ∼90° regardless of energy (Figure 12g) or pitch angle (Figure 12j).

There are a few points to be noted from Figure 12. First, we do not see evidence for electron drift resonance. At the resonance energy, log  *j*_e_ PSD would be peaked and the *E*_*ϕ*_‐ log  *j*_*e*_ and *B*_*μ*_‐ log  *j*_*e*_cross‐phase would exhibit a 180° shift (Claudepierre et al., 2013; Mann et al., 2013). Obviously, the waves were propagating westward (*m* > 0), opposite to the direction of the electron magnetic field gradient and curvature drift or, for the case of *m* > 0, the resonance energy was outside the energy range of the electron data examined, that is, *m* < 4 or *m* > 120.

To understand the cause of the *j*_e_ oscillations, we start from a general expression for the perturbation of particle phase space density

(14) $$\delta F=-M\delta B_{\mu }\frac{\partial F}{\partial M}-\delta W\frac{\partial F}{\partial W}-\delta L\frac{\partial F}{\partial L},$$

where F is a function of *L*, *M*, and *W* and *δW* and *δL* are the perturbations of *W* and *L* experienced by individual particles (Southwood, 1973). Both *δW* and *δL* depend on the spatial and temporal structure of the waves and on the energy and pitch angle of the particles. Therefore, *δW* and *δL* include effects of drift‐bounce resonances and need to be evaluated by taking an integral over the trajectory of the particle guiding center. Based on the absence of resonance signatures, we pay attention only to particle modulation mechanisms that do not include resonance effects.

**Figure 13:**
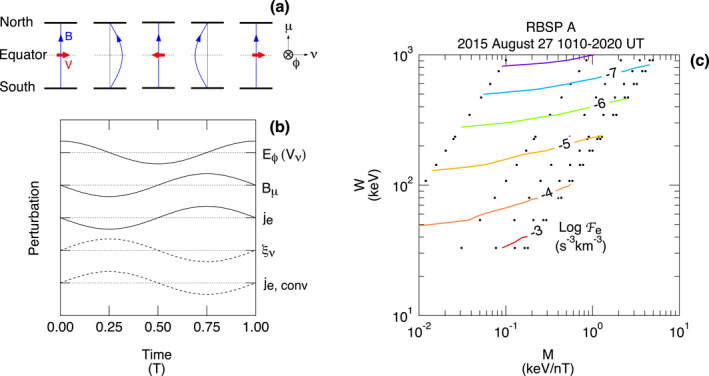
(a) Radial field line displacement pattern of a symmetric wave plotted a quarter‐wave period apart for one wave period (adopted from Figure 2a of Takahashi et al. [2011]). The red arrows indicate the plasma bulk velocity. (b) Schematic time series plots of the perturbations of observed field components (*E*_*ϕ*_ and *B*_*μ*_) and the electron flux (*j*_e_) during the symmetric wave event shown in Figure 12 along with the inferred field line displacement (*ξ*_*ν*_) and the electron flux perturbation associated with it (*j*_e, conv_). The phase of *j*_e, conv_ reflects the illustrated *ξ*_*ν*_ and the observed inward gradient of $F_{e}$. (c) $F_{e}$ derived from the *j*_e_ measurements made by RBSP A MagEIS at 1010–1020 UT, plotted as a function of *M* and *W*. The black dots indicate the data points corresponding to the energies and pitch angles of the measurements.

The first effect is radial convection of the gradient of the phase space density. The phase space density perturbation arising from the convection, denoted $\delta F_{c}$, is part of the *δL* term in Equation 14 (Southwood, 1973) and is given as

(15) $$\delta F_{c}=-\xi_{\nu }\frac{\partial F}{\partial L},$$

where *ξ*_*ν*_ is the radial component of field line displacement. This represents convection of the phase space density by the radial motion of the flux tube induced by *E*_*ϕ*_. Using the electron data shown in Figure 11, we obtained $F_{e}\left(M,W\right)$ at different time (*L*) steps and confirmed $\frac{\partial F_{e}}{\partial L}<0$. Here the independent variables are converted from *W* and pitch angle to *W* and *M* using the magnitude of the measured magnetic field. According to Figures 13a and 13b, *ξ*_*ν*_ associated with a symmetric wave reaches a maximum when the *E*_*ϕ*_ perturbation crosses zero from positive to negative. At the zero crossing, $\delta F_{e}$ (and the *j*_e_ perturbation) observed by a spacecraft reaches a maximum if $F_{e}$ has an inward gradient. This predicted *E*_*ϕ*_‐ log  *j*_e_ phase relationship is just the opposite of what is observed, and we exclude the convection of gradient as the cause of the observed *j*_e_ oscillations.

Another non‐resonance mechanism arises from the temporal variation of the magnetic field seen from drifting particles and is related to the *M* and *W* dependence of F. Kivelson and Southwood (1985) called this mechanism the betaton effect and expressed it as

(16) $$\delta F_{b}=-M\delta B_{\mu }\left(\frac{1}{B}\frac{\partial F}{\partial M}+\frac{\partial F}{\partial W}\right).$$

We can determine the relative importance of the two terms on the right‐hand side of Equation 16 using the contour display of $F_{e}$ in the *M*‐*W* space, shown in Figure 13c. The $F_{e}$ values for this figure are defined using the *j*_e_ data averaged over 1010–1020 UT (Figures 11d and 11e). We find $\frac{\partial F_{e}}{\partial M}>0$ and $\frac{\partial F_{e}}{\partial W}<0$ in the *M*‐*W* space covered by the MagEIS data. Because only the second derivative leads to $\delta F_{e}>0$ (in‐phase oscillations of *B*_*μ*_ and *j*_e_), we conclude that the second term on the right‐hand side of Equation 16 makes the dominant contribution to the *j*_e_ oscillations.

It is interesting to note that previous studies of compressional Pc5 waves at geostationary satellites reported antiphase *B*_*μ*_‐*j*_e_ oscillations when *j*_e_ had a pancake pitch angle distribution (Kremser et al., 1981; Takahashi et al., 1985). The first term on the right‐hand side of Equation 16 probably played the major role in modulating *j*_e_ during these Pc5 wave events.

To conclude the electron data analysis, we find no evidence of drift resonance with the symmetric waves. However, we caution the readers that we conducted the analysis of *j*_*e*_ oscillations for a time interval when the spacecraft was close to the outer *L* limit of the symmetric wave activity and detected an inward gradient of $F_{e}$. MagEIS data for lower *L* do not show clear *j*_e_ oscillations, and the electron response in that crucial region remains to be understood.

## 8 Discussion

**Figure 14:**
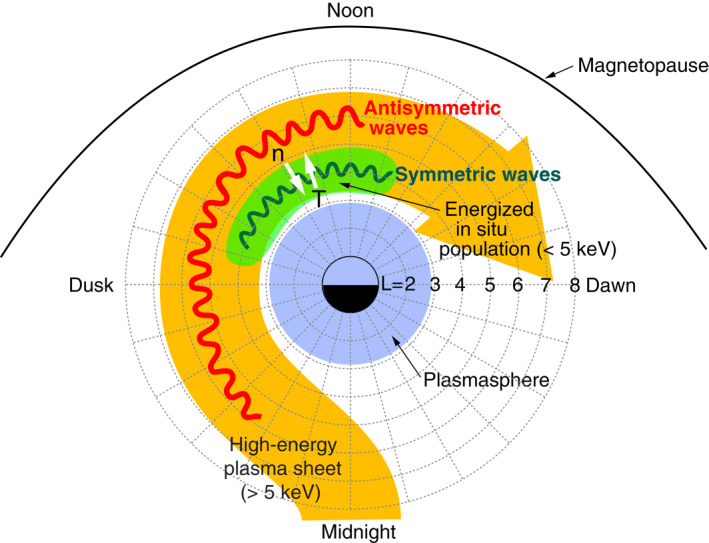
Illustration of stormtime ULF waves and their relation to ion populations incorporating results from previous and present studies. Antisymmetric waves are excited at *L* > 5 by energetic ions (high‐energy plasma sheet) drifting westward from the nightside injection region. Symmetric waves are excited at *L* < 5 in the region populated by lower‐energy ions (energized in situ population) in addition to the high‐energy plasma sheet population. In the region of overlap, the proton density (*n*) and temperature (*T*) have opposite gradients (white arrowheads). Both wave types are excited outside the plasmasphere.

The major finding of this study is that symmetric compressional waves were excited at 3.0 < *L* < 5.5 during a geomagnetic storm. Figure 14 provides a graphical description of this finding. The symmetric waves (wavy green curve) occur outside the plasmasphere (shaded blue) in the region where the energized in situ population (energies <5 keV, shaded green) overlaps the inner edge of the eastward‐drifting energetic ions (energies >5 keV, extending to >100 keV, shaded orange) that are injected from the plasma sheet. The in situ population has a higher density (*n*) and a lower temperature (*T*) than the injected energetic ions, leading to an inward density gradient and an outward temperature gradient when both populations are included in moment calculations. These gradients, which favor DCI, are colocated with the waves at *L* ≃ 3.0–5.0 in the example shown in Figure 9. We cannot determine the MLT extent of the symmetric waves because the MLT range covered by RBSP is limited in the present study. Therefore, the MLT span of the symmetric waves illustrated in Figure 14 could be very different from reality.

An important factor to be considered for the symmetric waves is that they occur in the region of *β* < 1. This is because the magnetic field is strong (*B* > 250 nT) at *L* < 5 so that the magnetic field pressure is higher than the ion pressure even when the high‐energy plasma sheet population and the energized in situ population are simultaneously present. Note that the latter population does not contribute much to *β* because it appears at energies lower than the core of the pressure‐carrying ring current population (Williams, 1981). The moderate *β* value makes the DMI an unlikely mechanism for the symmetric waves. This warrants a different theoretical approach to the symmetric waves compared to that for compressional Pc5 waves observed at *L* > 5. In addition to DCI, DRI is a possible mechanism to excite the symmetric waves.

The symmetric waves are distinct from antisymmetric poloidal waves (wavy red curve) routinely detected at *L* > 5 primarily on the afternoon side (Anderson et al., 1990; Kokubun, 1985) often with a very strong magnetic field compressional component. The energy source of the antisymmetric waves is believed to be the injected energetic ions. The ions occupy a region extending to *L* > 7, explaining why the waves are excited beyond geosynchronous orbit. For the antisymmetric waves, there is an established theoretical framework, which incorporates ring current ion pressure, coupling between Alfvén and compressional modes, and effects of bouncing particles (Chen & Hasegawa, 1991; Cheng & Lin, 1987; Southwood, 1976). The free energy sources for the waves include a bump‐on‐tail energy distribution (Takahashi, Oimatsu, et al., 2018) and a radial gradient of the phase space density (Oimatsu et al., 2018). Quantitative tests of the generation mechanisms are possible for the antisymmetric waves because observations provide not only the relevant background plasma parameters but also the phase and amplitude relationship between the wave fields and the perturbed plasma pressure and ion fluxes (Kokubun et al., 1989; Kremser et al., 1981). In addition, it is possible to determine *m* through interspacecraft phase delay analysis (Takahashi et al., 1985) or using ion finite Larmor radius sounding techniques (Lin et al., 1988; Min et al., 2017). In future studies, we will look for symmetric waves to which we can apply these techniques to better specify the wave properties. Knowledge of *m* is also necessary to understand the response of electrons to the waves.

Whether the energized in situ population is indeed a prerequisite for the symmetric waves needs to be determined by analyzing more events. The population was present on the few RBSP equatorial passes with symmetric waves during the selected geomagnetic storm. Whether or not this is a coincidence can be determined by a statistical analysis of stormtime ULF waves encountered by RBSP over the mission period of 7 years. We note in Figure 2 that the first two symmetric wave events highlighted (orbits 2920 and 2922) occurred a few hours after very strong substorm activity (AL ≤ − 1,000 nT) and that the third event also occurred ∼8 hr after another (less intense) substorm. In addition, the wave power in those three symmetric ULF wave cases seems to correlate to the length of time passed since the previous substorm. By contrast, there is no such substorm activity before the antisymmetric waves observed on the fourth highlighted event in Figure 2. The appearance of the in situ population and the symmetric waves could be related to the history of substorm activity. The high‐energy plasma sheet population is obviously caused by ion injection during substorms, and plasma waves excited by this population might play a role in the formation of the in situ population.

## 9 Conclusions

We have studied ULF waves in the inner magnetosphere excited during a moderate geomagnetic storm. Analysis of RBSP and ground magnetometer data revealed the following properties of the waves:

1. The waves are excited at *L* = 3–5.5 outside the plasmasphere.
2. At the magnetic equator, the waves produce strong perturbations in *E*_*ν*_, *E*_*ϕ*_, and *B*_*μ*_ but not in *B*_*ν*_ or *B*_*ϕ*_, consistent with a symmetric mode structure about the equator.
3. The waves are detected in the region of ion flux enhancement at energies lower than ∼5 keV.
4. Electron fluxes at energies from ∼30 keV to ∼1 MeV oscillated in phase with *B*_*μ*_ regardless of the pitch angle.

We also discussed possible excitation mechanisms for the symmetric waves:

1. The threshold for DMI is not met.
2. The phase space density of protons shows an inward gradient at some energy, which could drive DRI if the waves have a very high azimuthal wavenumber.
3. Proton bulk parameters show properties consistent with DCI.

## Data Availability

The data used in this study are publicly available from the following sources: NASA Goddard Space Flight Center (GSFC) Space Physics Data Facility Coordinated Data Analysis Web (https://cdaweb.gsfc.nasa.gov/index.html) for RBSP data; NASA/GSFC Space Physics Data Facility OMNIWeb Plus (https://omniweb.gsfc.nasa.gov/) for Solar wind OMNI data; Zenodo archive (https://doi.org/10.5281/zenodo.5639728) for EMMA data; and World Data Center of Geomagnetism, Kyoto (http://wdc.kugi.kyoto-u.ac.jp/) for geomagnetic activity indices.
